# Analysis of current methods and Welfare concerns in the transport of 118 horses by commercial air cargo companies

**DOI:** 10.1186/s12917-024-03999-9

**Published:** 2024-04-26

**Authors:** Martina Felici, Naomi Cogger, Leonardo Nanni Costa, Christopher Bruce Riley, Barbara Padalino

**Affiliations:** 1 Department of Agricultural and Food Sciences, University of Bologna, Bologna, Italy; 2 School of Veterinary Science, Massey University, Palmerston North, New Zealand; 3 Ontario Veterinary College, University of Guelph, Guelph, Canada

**Keywords:** Equine, Air transport practices, Flight, Behavior, Health, Welfare

## Abstract

**Background:**

Studies on equine air transport practices and consequences are scarce. This prospective study aimed to describe horse and air journey details and practices, document how horse behavior and health changed during the air transport phases, quantify the occurrence of welfare issues, and identify possible associations between horse and journey details, air transport practices, and welfare issues.

**Results:**

Data were collected from before departure to five days after arrival on 118/597 horses traveling on 32 commercial air journeys on different routes, varying in duration and conditions. Most horses were middle-aged warmblood females, 26% of which were pregnant, and being moved by air for sales. Before flying, most were quarantined (median: 18; IQR: 9–53 days), and their fitness for travel was certified by veterinarians. At the departure airports, external temperatures varied from − 6 °C to 33 °C, and horses were loaded by experienced flight grooms (median: 35; IQR: 15–40 years) into jet stalls (three-horse: 87%, two-horse: 13%). During the flights, horses were regularly watered (water intake median: 14 L) and fed *ad libitum* (feed consumption median: 8 kg). At the arrival airport, horses were unloaded from the jet stalls, and external temperatures ranged from − 5 °C to 32 °C. Then, all horses were transported to arrival quarantine by road. Air transport phases affected horses’ health status and behavior; increased heart and respiratory rates and behaviors, such as pawing, head tossing, and vocalization, were mainly identified at departure and arrival. Horse interaction, nasal discharge, increased capillary refill time (CRT), and abnormal demeanor were observed more often one hour before landing while resting and normal capillary refill time were more often displayed five days after arrival (all *P* < 0.01). One hour before landing, horses with bad temperament and horses of unknown temperament were more likely to develop nasal discharge when transported in winter and autumn (*P* < 0.001). The likelihood of an increased CRT was associated with shorter flights in horses of unknown travel experience (*P* < 0.001). Ten horses were injured, and 11 developed pleuropneumonias (i.e., shipping fever).

**Conclusions:**

Air transport is a complex procedure with several different phases affecting horse health and behavior. Therefore, experienced staff should carefully manage each horse before, during, and after air journeys to minimize welfare hazards.

**Supplementary Information:**

The online version contains supplementary material available at 10.1186/s12917-024-03999-9.

## Background

Horse air transport is increasingly popular, especially for moving high-performance and high-value horses [1]. Traveling by air, horses can more easily participate in international competitions or be transferred for breeding worldwide [2]. In addition, air transport covers equal or greater distances in less time and is considered less stressful than road transport [3]. However, air transport is a complex event consisting of ground and air phases [4], and international air transport often includes a quarantine period before or after the air journey [5]. Quarantine is intended to limit the spread of infectious diseases, and procedures vary among countries depending on the biosecurity risk and specific regulatory requirements on disembarkation [6, 7]. Although critical for biosecurity, quarantine isolation can be a stressor (e.g., change of environment, social isolation) to the horse [2]. Road transport is another ground phase carried out as part of air transport, especially when horses are moved to quarantine facilities and departure airports in the country of origin and from arrival airports to quarantine facilities in the destination countries [4]. Therefore, air transport in its entirety includes the critical factors associated with road transport, such as handling, loading in the vehicle, confinement, vibration, and unloading from the vehicle [2]. Another crucial and potentially welfare-compromising phase is handling the horses at the departure airport, including loading them into the jet stalls and maneuvering them and the horses contained within toward the side of the aircraft. Dedicated vehicles bring one or more jet stalls in trains to the scissor lift that raises them to the height of the aircraft door, thereby causing vibrations and movement, possibly eliciting sensory overstimulation [4, 5]. Finally, air transport is often accompanied by technical stops for re-fueling *en route*, the re-arrangement of goods in the cargo hold, and occasionally flight transfers [4]. During aircraft stops, horses may be kept onboard or unloaded and reloaded, exposing them to temperature changes and motions that can present as additional stressors [4].

During the flight, some transitional events are recognized to be critical. For example, horses experience increased heart rates during takeoff, landing, and in-flight turbulence and express social behaviors such as aggression and submission towards conspecifics [3, 8]. Moreover, several heart rate variability parameters shifted during air transport, indicating sympathetic activation [9]. Thus, although it is reported that during the flight, horses are better adapted to air transport than to road transport [8], this air journey phase can be stressful due to potential welfare-compromising hazards, such as limited space, water and feed withdrawal, extreme microclimate conditions, and journey duration [5, 10].

Air journeys are usually prolonged (> 8 h) [5], exposing horses to the risk of welfare consequences, including sensory overstimulation, fever, weight loss, dehydration, thermal stress, and respiratory disease, including pleuropneumonia (i.e., shipping fever) [5, 8, 11–13]. Long-distance transport of horses by air has also been associated with coughing, anorexia, nasal discharge, swollen and painful lymph nodes, sweating, distal limb swelling and increased serum amyloid A (i.e., an inflammatory marker) [14]. The cited studies have focused on race and sport horses.

Given the complex nature of air transport, its increasing use in the equine industry, and the possible associated welfare risks to which horses are subjected, specific guidelines have been developed [6, 7]. The International Air Transport Association (IATA) regulates live animal transport by Live Animals Regulations, specifying container requirements and good practices to be performed during loading and air transport [15]. Guidelines based on accepted best practices provide a helpful starting point. However, these lack underpinning scientific evidence, as few studies have reported the horse’s physiological and behavioral responses to air transport practices. Therefore, data describing the practices and the health and welfare consequences related to air transport is needed to inform practice [5].

The authors of the current work hypothesized that the behavior and health status of the horses change during the air transport phases, and that horse (i.e., temperament, sex, experience/training in traveling) and journey (e.g., flight duration, stops) details, and air transport practices (e.g., watering, feeding in transit) may predispose them to transport-related behavioral and health problems (i.e., welfare issues). This study aimed to describe horse and air journey details and common air transport practices, to document how horse behavior and health status change during the different air transport phases, and to quantify the incidence of associated injury, health, and behavioral problems (i.e., welfare issues). The authors also sought to identify possible associations between horse and journey details, air transport practices, and welfare issues.

## Results

Four previously contacted shipping companies (i.e., companies that organize the logistics of the transport of horses by air) that were members of the Animal Transportation Association (ATA), collected data on 32 journeys from 2020 to 2023, during which a total of 597 horses were transported. Data were collected on a sample of 118 of these horses using a standard protocol composed of seven blocks of questions (Table S1, Additional File 1). The completion rates by participants varied across different question blocks. The second block had the highest completion rate (93%), followed by the third (92%), fourth (86%), and first (85%) blocks. The fifth block had the lowest completion rate (58%), followed by the seventh (59%) and sixth (64%) blocks.

### Air journey details

**Fig. 1:**
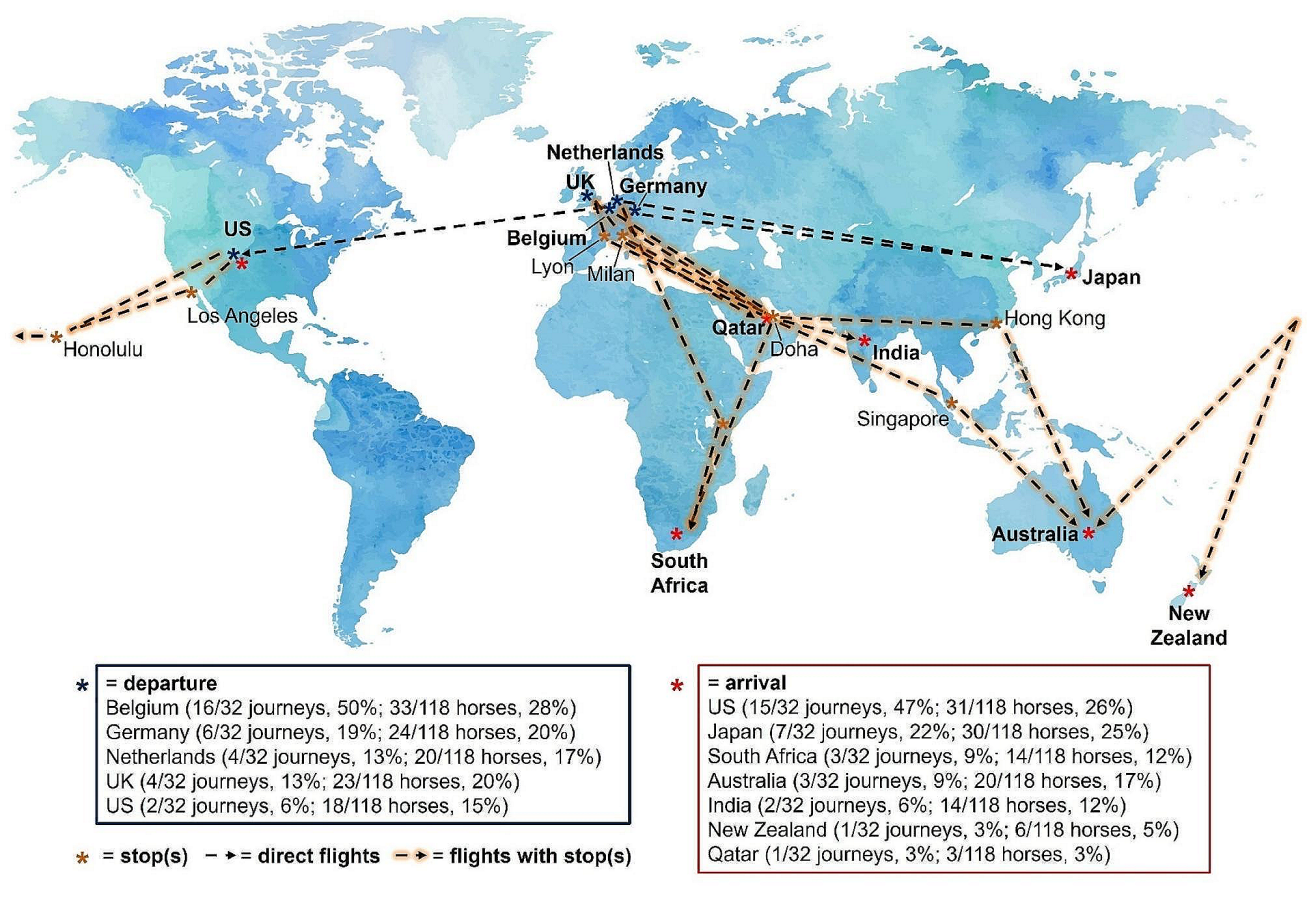
Routes and stop(s) of the 32 international air journeys transporting 118 horses monitored (2020–2023) US: United States; UK: United Kingdom

**Fig. 2:**
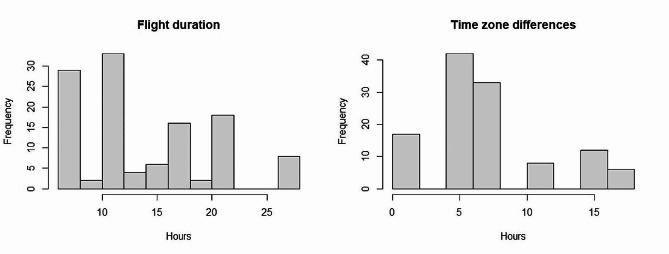
Histogram of flight duration (left) and time zone differences (right) of 32 air journeys monitored

Table 1 shows details of the 32 air journeys included in this study. The journeys monitored were distributed throughout the year but mainly occurred during winter (Winter: 45%; Spring: 19.5%; Summer: 19.5%; Autumn: 16%), according to the location of departure, which were all in the Northern hemisphere. For air journeys, a median of 14 (IQR: 12–21) horses were transported, and a median of four horses (IQR: 3–6) per trip was closely monitored. Horses traveled with six different air cargo companies (i.e., contracted by the four shipping companies), transported by wide-body aircraft (i.e., Boeing 747 or Boeing 777), on flights that were most often used for freight (98%; 115/117 horses). Twenty-two flights (22/32 journeys, 69%; 61/118 horses, 52%) were direct, while the others had several stops (i.e., five flights/one stop, four flights/two stops, one flight/three stops). For these, the median stop duration was 3 h 30 min (IQR: 1 h 45 min − 5 h) (Fig. 1). Four were technical stops for loading air cargo with or without unloading/loading the jet stalls, while for the other six flights, there was an aircraft change requiring the jet stalls to be moved. In only two cases, horses were unloaded from the jet stalls and rested at an animal lounge (i.e., an animal facility at the airport where experienced staff cared for, fed, and sheltered the traveling horses). During the stops, the horses were exposed to different external environmental temperatures, varying from 3 °C to 42 °C, depending on season and routes. However, when the horses stayed in the cargo hold during these stops, the air temperature was not controlled and varied from 12.0 °C to 21.5 °C. The median duration of the flights was 11 h 30 min (min-max: 7–28 h; IQR: 8 h 15 min − 16 h 30 min) (Fig. 2), including delays, which varied from 1 h to 3 h 30 min for three journeys. The median time zone difference between the country of departure and arrival was 7 h (min-max: 1–18 h; IQR: 5 h 30 min – 16 h 30 min) (Fig. 2).

**Table 1:** Details of the 32 international air journeys transporting 597 horses (2020–2023)

Journey ID	Route	Journey month	Journey season	Boeing aircraft model	Departure-Arrival T range (C°)	N° horses in the cargo	N° horses monitored	Flight duration*
1	NL-JP	May	Spring	747 F	14.0–18.0	14	6	15 h
2	DE-JP	November	Autumn	777–200 F	5.0–14.0	15	5	11 h 30 min
3	DE-JP	December	Autumn	777–200 F	8.0–6.0	13	5	11 h 30 min
4	DE-JP	January	Winter	777–200 F	-2.0–10.0	21	4	11 h 30 min
5	DE-JP	February	Winter	777 F	15.0–11.0	14	4	11 h 30 min
6	DE-JP	March	Winter	777 F	8.0–15.0	16	4	11 h 30 min
7	DE-JP	September	Summer	777 F	20.0–28.5	15	2	11 h 30 min
8	BE-US	August	Summer	747	17.7–28.0	15	2	7 h
9	BE-US	August	Summer	747 F	15.5–26.1	12	1	8 h
10	NL-ZA	August	Summer	777 F	22.8–21.0	14	4	16 h 30 min
11	NL-ZA	January	Winter	747 F	7.2–23.0	21	6	11 h 30 min
12	BE-US	March	Winter	747–400 F	9.0–14.0	29	2	7 h
13	BE-US	April	Spring	747–400 F	7.0–13.6	30	2	7 h
14	BE-US	April	Spring	747–400 F	10.0–14.1	21	2	7 h
15	BE-US	April	Spring	747–400 F	12.2–14	30	1	7 h
16	BE-US	May	Spring	747–400 F	12.0–13.6	30	2	7 h
17	BE-US	May	Spring	747–401 F	17.0–18.0	30	2	8 h 15 min
18	BE-US	June	Spring	747–400 F	16.0–23.8	12	2	7 h
19	NL-ZA	August	Summer	747 F	33.0–16.0	27	4	13 h 30 min
20	UK-AU	August	Summer	777 F	14.0–6.0	17	6	28 h
21	BE-AU	September	Summer	777–300 F	24.0–16.0	38	2	28 h
22	BE-US	October	Autumn	747–400 F	10.0–14.4	21	3	7 h
23	US-NZ	November	Autumn	747–400 F	0.0–20.0	6	6	21 h 35 min
24	BE-US	January	Winter	747 F	4.0–2.5	23	2	7 h
25	UK-IN	January	Winter	777 F	-6.0–22.0	12	12	16 h 30 min
26	BE-US	February	Winter	747 F	2.0–13.3	21	1	7 h
27	BE-US	March	Winter	747 F	4.0–5.0	30	2	7 h
28	BE-US	March	Winter	747–400 F	9.0–10.0	12	4	7 h
29	US-AU	March	Winter	747–400 F	-4.0–18.0	12	12	22 h
30	BE-US	April	Spring	747–400 F	7.0–12.0	21	3	7 h
31	UK-QA	April	Spring	777 F	8.0–27.5	3	3	11 h 30 min
32	UK-IN	July	Summer	777 F	13.0–32.0	2	2	20 h

NL: The Netherlands; JP: Japan; DE: Germany; BE: Belgium; US: United States; ZA: South Africa; UK: United Kingdom; AU: Australia; NZ: New Zealand; IN: India; QA: Qatar

h: Hour(s)

min: Minutes

Boeing aircraft model: The “F” at the end of the aircraft model number stands for the “freight” version of that model

* Flight duration was calculated by adding the duration of the flight (from take-off to landing) to the duration of any stops and delays

### Owner and horse details

Table 2 describes the owners’ and transported horses’ details (see Tables S2 and S3 of Additional File 1 for more information about the names and definitions of all variables). In instances when agents could not establish contact with the owner or retrieve the history of some horses, particularly those acquired through auctions, they selected the “I do not know” option. For the purposes of reporting, the responses were later recorded as “unknown”. Most owners who transported their horses by air were involved in equine business (i.e., people involved with horses for financial reward; e.g., breeders), with a median of 30 years of experience in the equine industry (min-max: 3–55 years; IQR: 15–30 year; data missing: 12 (10%)), and usually custodians of a median of 29 horses (min-max: 0–75; IQR: 18–40; data missing: 12 (10%)) on their business properties.

The horses in the study were reported by their owners to usually have a “good temperament” scarce previous road travel experience and were rarely trained to travel by habituation and other unspecified methods. Despite this, most horses were reported to be “easy to load”, and few Transport-Related Problem Behaviors (TRPBs) were reported. Among them, five horses were previously observed to display kicking behavior, four showed signs of anxiety, two exhibited refusal to load, and one horse each displayed freezing, scrambling, rearing, and escape behaviors. Additionally, four horses were categorized under “other” behaviors (i.e., attempting to lie down, tail rubbing). Of the 12 horses with TRPBs, seven exhibited them during loading, five during preloading, five during traveling, two during unloading, two post-travel, and the road transport stage during which the TRPB occurred was unknown for one. Two horses had a history of Transport-Related Health Problems (TRHPs): one had colic, and the other had an injury and muscular problems.

Data regarding treatments performed one week before the air journey monitored were unknown for 40% of the horses. Among those known, 58 horses received no treatment before the journey. Of the remaining 13, six horses were covered with rugs, four had their shoes removed, and four had bandages or boots applied to the distal limbs. Additionally, one horse each received antibiotics, tranquilizers, vaccinations, fluids, and other forms of treatment.

Sampled horses were most frequently middle-aged (median: 7 years; min-max: 1–17 years; IQR: 4–9 years; data missing: 9 (8%)), warmblood females shipped from the United Kingdom and transported for sales. Among the 57 transported mares, 15 were pregnant (15/57 horses, 26%). Of the 53 male horses, 11 were stallions, and 42 were geldings. Eleven horses were between eight months and two years old.

**Table 2:** Descriptive statistics of owner and 118 horse details, including the past travel history before air transport (2020–2023)

Variable name	Category	Number	Percentage
**Horse owner details**			
Owner’s equestrian sector	Show jumping	32	27
	Breeding	19	16
	Other	17	14
	Dressage	14	12
	Recreational riding	11	9
	Thoroughbred racing	6	5
	Showing	3	3
	Western	3	3
	Pony club	1	1
	Unknown	12	10
Relationship with the horse	Professional	96	81
	Amateur	10	8
	Unknown	12	10
**Horse details**			
Horse sex (*n* = 110) ^a^	Female	57	52
	Male	53	48
Horse breed (*n* = 110) ^a^	Warmblood	60	55
	Thoroughbred	25	23
	Quarter Horse	8	7
	Other	17	15
Country of stable of departure (*n* = 110) ^a^	United Kingdom	42	38
	Belgium	27	25
	USA	16	16
	Germany	11	10
	Other	14	13
Reason for moving the horse by air (*n* = 110) ^a^	Sale	59	54
	Breeding	22	20
	Competition	13	12
	Personal relocation	10	9
	Racing	6	5
**Horse past travel history**			
Horse temperament profile	Bad	20	17
	Good	64	54
	Unknown	34	29
Horse experience in road travel	Few trips	37	31
	Travel regularly	34	29
	Unknown	47	40
Horse training to travel	Untrained	74	63
	Trained	20	17
	Unknown	24	20
Horse loading in a vehicle	Easy	77	65
	Resistant	21	18
	Unknown	20	17
Horse previous Transport-Related Problem Behaviors	Yes	12	10
	No	85	72
	Unknown	21	18

Total percentages: Note that total percentages do not always sum to 100% for every characteristic due to rounding

^a^ Missing data: Data were missing for some variables, and the denominator for percentage calculations is based on the number of horses for which data were available. The total number of horses with available data is indicated next to each variable descriptor. Data were missing for Horse sex, Horse breed, Country of stable of departure, and Reason for moving the horse by air (missing = 8 horses, 7%)

### Air transport practices

#### Practices from the stable of origin to the departure airport

Table 3 describes the management practices performed in quarantine before departure. All horses were transported by road, mainly via commercial operators, to quarantine facilities. A quarter of the horses only transited through facilities and spent less than one day there, while most stayed in quarantine for several days. The median quarantine duration was 18 days (min-max: 9–53 days; IQR: 11–30 days; data missing: 36 (31%)). The median number of horses housed in the same quarantine barn was 10 (min-max: 3–20; IQR: 6–12; data missing: 8 (7%)). During the quarantine period, most horses were exercised at least once a day for a median of 1 h 45 min (min-max: 30 min–24 h; IQR: 45 min–9 h; data missing: 26 (22%)). Before leaving quarantine, all the horses were examined by a veterinarian, and all but two were deemed fit for travel. The two horses classed as unfit for travel had health issues: abortion and fever. Therefore, these horses did not travel and were not included in the statistical analyses. The horses deemed fit for travel were transported by road to the departure airport, mainly by commercial operators (113/118 horses, 96%) using large trucks (110/113 horses, 97%). The median distance traveled to the airport was 195 km (min-max: 18–697 km; IQR: 48–490 km; data missing: 8 (7%)), and arrival at the airports was most often at night or in the evening.

**Table 3:** Descriptive statistics of the management practices during quarantine before departure for air transport of 118 horses (2020–2023)

Variable name	Category	Number	Percentage
Quarantine	Yes	90	76
	No	28	24
Exercise in quarantine (*n* = 110) ^a^	Yes	98	89
	No	12	11
Type of exercise (*n* = 108) ^a^	Loose in a paddock	71	66
	Horse walker	41	38
	Riding under saddle	1	1
	Other	5	5
Exercise frequency per day (*n* = 104) ^a^	None	12	12
	Once	88	85
	Twice	4	4
Arrival at departure airport (*n* = 110) ^a^	Morning	18	16
	Afternoon	23	21
	Evening	27	25
	Night	42	38

Total percentages: Note that total percentages do not always sum to 100% for every characteristic due to rounding

^a^ Missing data: Data were missing for some variables, and the denominator for percentage calculations is based on the number of horses for which data were available. The total number of horses with available data is indicated next to each variable descriptor. Data were missing for Exercise in quarantine and Arrival at departure airport (missing = 8 horses, 7%), Type of exercise (missing = 10 horses, 8%), and Exercise frequency per day (missing = 14 horses, 12%)

#### Practices at departure airports

**Fig. 3:**
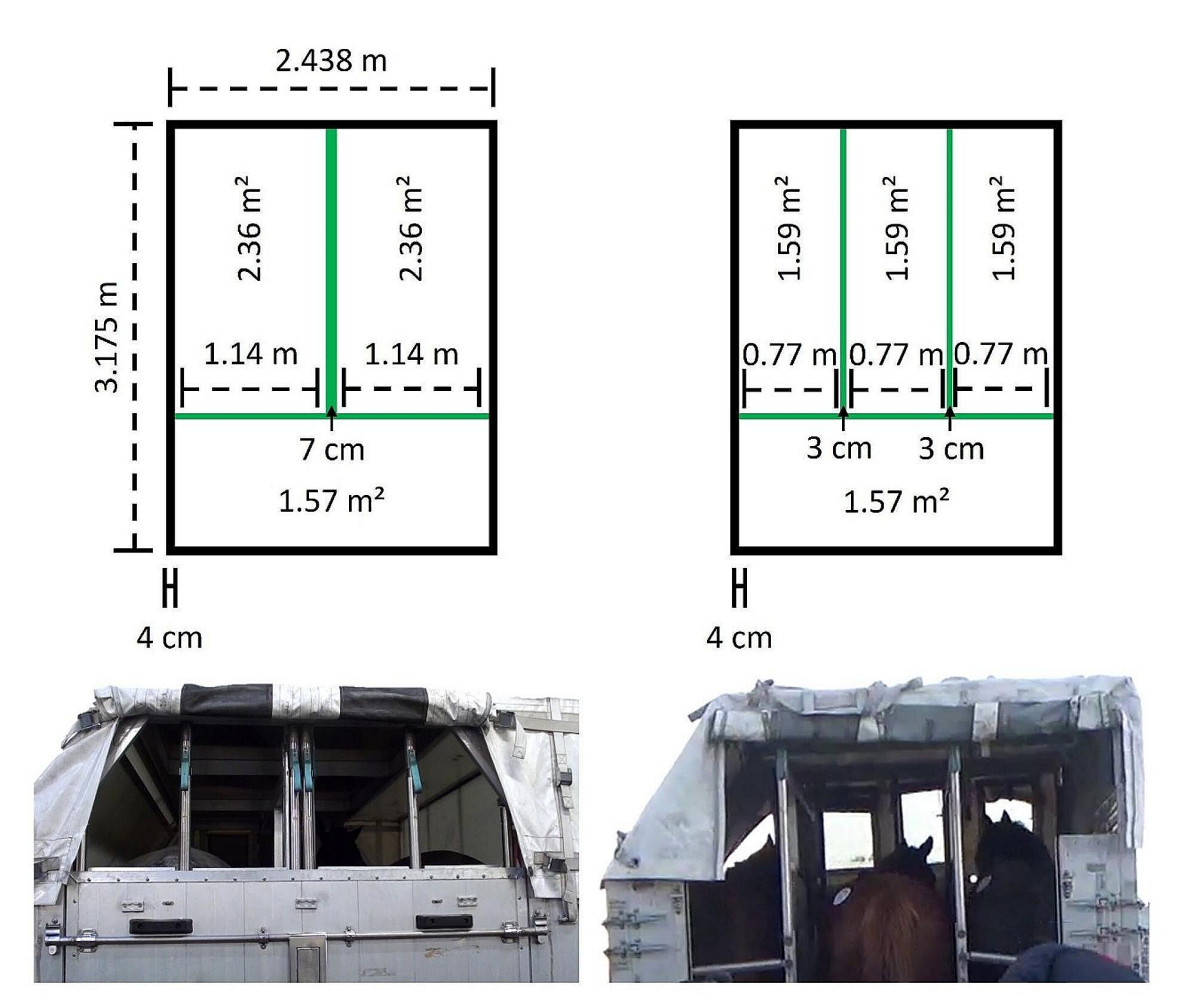
Top (diagrams) and rear (photos) views of standard jet stalls and relative space when two (left) or three (right) horses are accommodated inside it

At departure airports, the horses waited in the road transport vehicle for a median of 1 h 53 min (min-max: 5 –6 h 15 min; IQR: 52 min–2 h 15 min; data missing: 38 (32%)) before being unloaded by experienced flight grooms (median experience in horse handling: 35 years; min-max: 7–60 years; IQR: 15–40 years; data missing: 9 (8%)). The flight grooms visually checked the health status of the horses. All were judged fit for transport and loaded into jet stalls (Additional File 2). Ten horses (10/102, 10%) were equipped with supplies, including three horses with rugs and distal limb bandages, two each with bell boots and travel boots, one with a rug only, one with bandages only and one with a tail bandage only. Most were described as easy to load (63/78 horses, 81%), but some showed hesitation (13/78 horses, 17%), and two had to be pushed (2/78 horses, 3%) during loading procedures. Horses were most frequently accommodated in three-horse capacity jet stalls (102/117 horses, 87%), bedded with shaving (101/117 horses, 86%) or pellets (16/117 horses, 14%). The jet stalls consisted of light alloy frames mounted on a steel base with adjustable glass reinforced polyester coated partitions. The floor of the jet stalls was covered with non-slip rubber, and the interior of the jet stalls had adjustable openings (i.e., plastic curtains) to control light and airflow. Roofs were designed for wide-body aircraft, with left, right or double-contoured roofs. Standard-size jet stalls were 317 cm long, 243 cm wide and 243 cm high. The walls were usually 4 cm thick, and the partitions 3 cm thick, so the usable width was 228 cm in a two-horse jet stall (114 cm per horse) and 229 cm in a three-horse jet stall (77 cm per horse). Most horses thus had a space allowance of about 1.59 m^2^ (77 cm wide*206 cm length) up to the chest bar, and a space free for the head and neck shared with the other horses. However, this space (about 0.5 m^2^/each) was filled with hay nets and other types of equipment (e.g., water tanks, buckets, and saddles) (Fig. 3).

The horses remained in the jet stall for a median of 3 h (min-max: 1 h 30 min – 5 h 30 min; IQR: 2 –4 h; data missing: 32 (27%)) before loading into the aircraft (Additional File 3). In total, the horses stayed at the airport for a median of 5 h (min-max: 2 h 30 min – 11 h 45 min; IQR: 4 –5 h 45 min; data missing: 15 (13%)). Flights departed most frequently at night (47/112 horses, 42%) or in the morning (47/112 horses, 42%) but rarely in the evening (10/112 horses, 9%) or afternoon (8/112 horses, 7%).

#### Practices during the flight

**Fig. 4:**
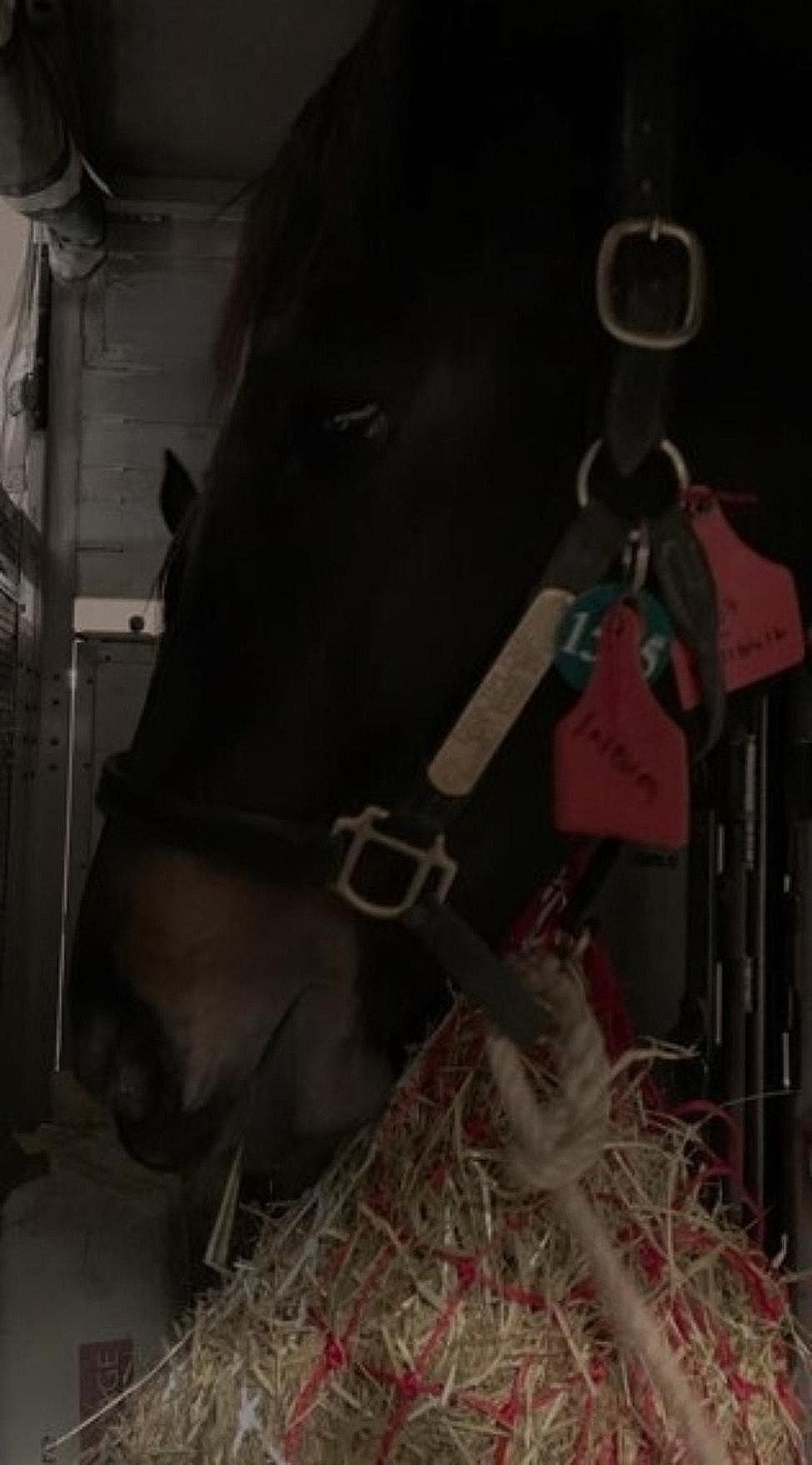
Low light conditions of a horse traveling by air in a three-horse jet stall towards India

**Fig. 5:**
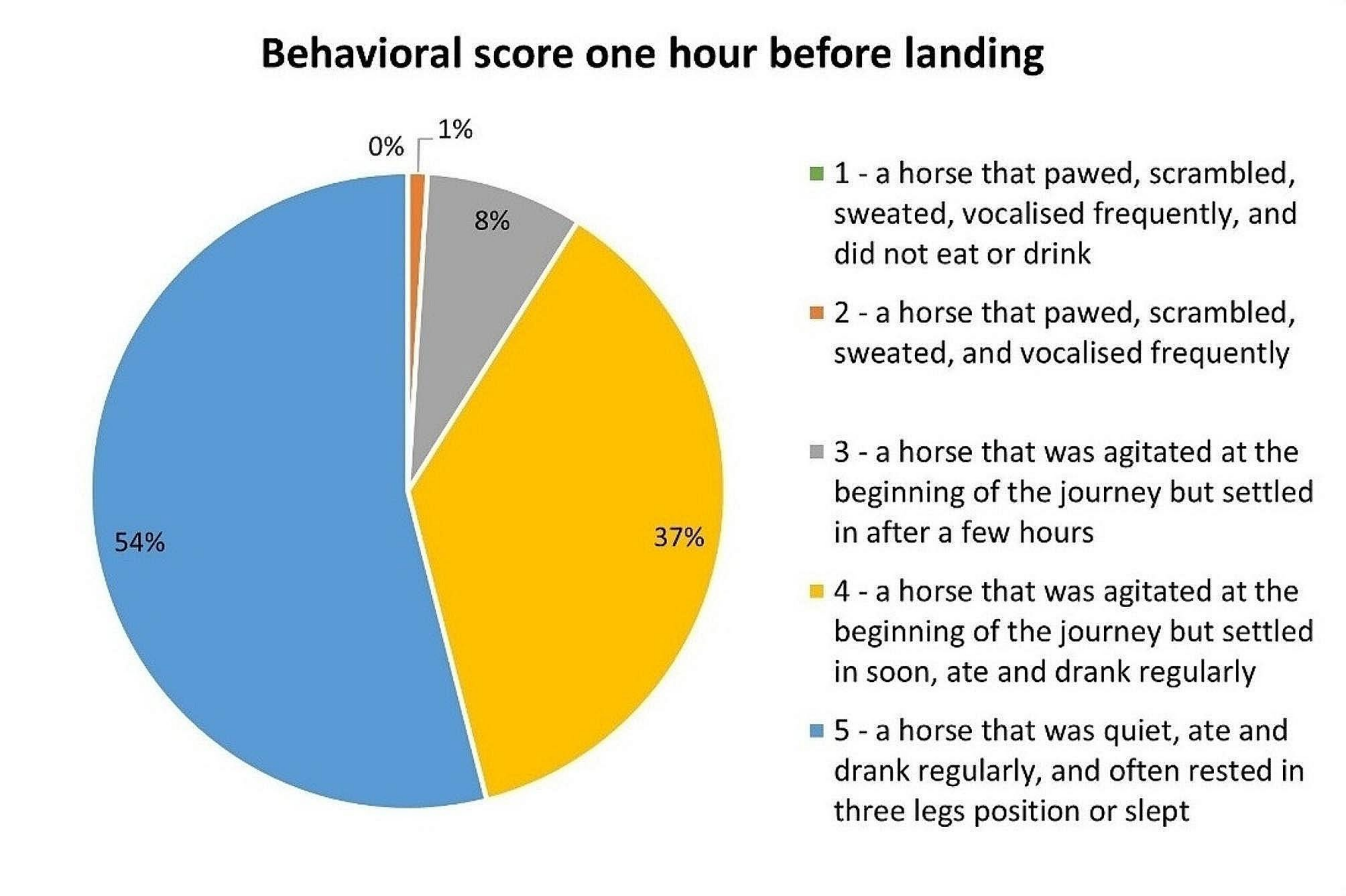
Behavioral scores were given to the horses by the flight grooms one hour before landing Data were available for 100 horses transported by air (missing = 18 horses, 15%)

The jet stalls were usually placed at the extremities of air cargo holds (i.e., before or behind wings) (60/118 horses, 54%), with the holds most frequently filled to above 80% of their capacity with jet stalls and other air cargo pallets (89/118 horses, 80%). Jet stalls did not have a light source inside, and the cargo hold light was soft (Fig. 4). Consequently, the flight grooms had to use bright LED flashlights to check horses (Additional File 4). During the flight, the horses were usually watered regularly (two hourly: 46/117 horses, 39%; or three hourly: 59/117 horses, 50%) and rarely *ad libitum* (12/117 horses, 10%). Feed (grass hay: 97/117 horses, 83%; haylage: 20/117 horses, 17%) was offered *ad libitum* and was positioned in most jet stalls at nostril level (89/117 horses, 76%). Horses consumed food and water, except for one that drank but did not eat, and another that ate but did not drink. A median of 8 kg (min-max: 4–30 kg; IQR: 7–14 kg; data missing: 33 (28%)) of feed was consumed. The median water intake was 14 L (min-max: 2–50 L; IQR: 5–24 L; data missing: 25 (21%)), but horses often did not drink during the initial (i.e., first-third) round of watering. Three very agitated horses (3/103 horses, 3%), who continued to scramble, kick, and vocalize, received sedation during the flights (i.e., one received acepromazine administered orally, for the other two details were not reported). Eight horses, including the one sedated, received an oral paste of electrolytes and vitamins. The horses traveled with their head tied on a long line or untied (82/111 horses, 74%) or tied short (29/111 horses, 26%). Most horses (91/111 horses, 90%) traveled near quiet neighbors, while ten horses (10/111 horses, 10%) were reported to travel near nervous neighbors. Using a behavioral score ranging from 1 to 5 (Fig. 5), most horses were scored by flight grooms as 5 or 4.

#### Practices on arrival

All horses were unloaded from the aircraft by the flight grooms, who sometimes stayed inside the jet stalls to reassure and calm them (Additional File 4). After this, all documents and the horses were transferred to the hands of other shipping agents/grooms/airport officers, who moved them to an animal lounge or immediately loaded them into a road transport vehicle for transfer to the quarantine facility. At the facility, horses were checked by veterinarians or quarantine officers.

#### Environmental parameters during the air transport phases

**Fig. 6:**
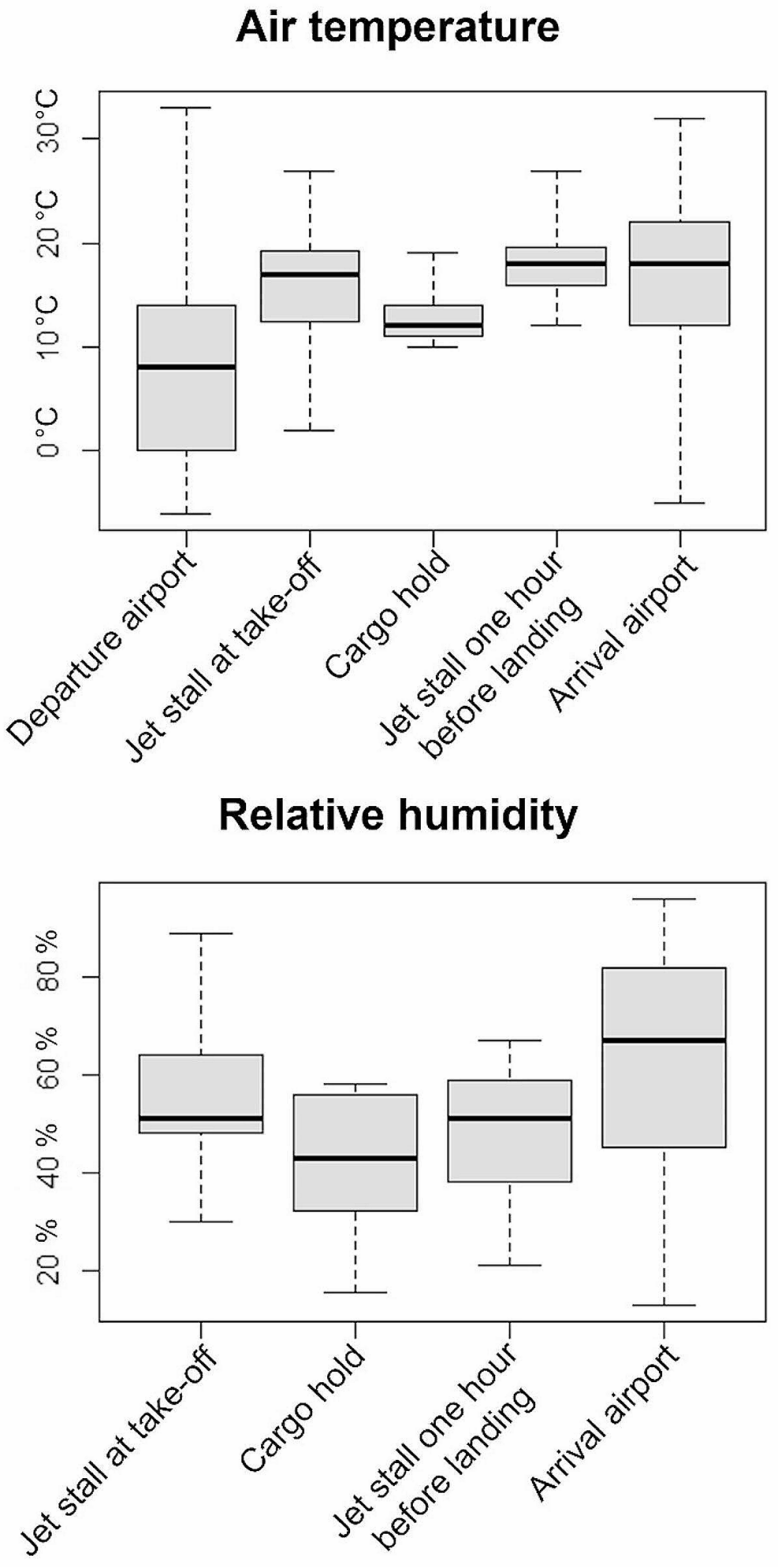
Air temperature and relative humidity during the different air transport phases ^a^ Missing data: Data were missing for some variables, and the numerical descriptive statistics are based on the number of horses for which data were available. Data were missing for T in the cargo hold one hour before landing (missing = 17 horses, 14%), T in the jet stall one hour before landing (missing = 39 horses, 33%), T in the jet stall at take-off (missing = 43, 36%), H in the cargo hold one hour before landing (missing = 51 horses, 43%), H in the jet stall one hour before landing (missing = 62 horses, 53%), H in jet stall at take-off (missing = 65 horses, 55%). Median = band near the middle of the box, 25th percentile = bottom of the box, 75th percentile = top of the box, minimum = lower whisker, maximum = upper whisker

Box plots of the air temperature (T) and relative humidity (H) data for the different air transport phases are displayed in Fig. 6. For monitored journeys, the T at the departure airport varied the most (min-max: -6.0 °C and 33.0 °C), affecting the micro-environment of the jet stalls immediately after loading (T min-max: 2.0 –27.0 °C; H min-max: 30–89%). The peak of 89% H in the jet stall was recorded on a journey departing from the United Kingdom in August, in which horses stayed for 1 h 30 min before departure. T inside the cargo hold was controlled and usually remained between 10.0 °C and 14.0 °C. Therefore, T of the jet stall one hour before landing varied less, from 12.0 °C to 27.0 °C. H in the cargo hold was not artificially controlled, varying from 16 to 58%, and that recorded in the jet stalls varied similarly from 21 to 67%. Jet stall values were always higher than those of the cargo hold, as the median difference of T and H between jet stalls and the cargo hold was 4 °C (IQR: +3.0 – +6.0 °C; data missing: 48 (41%)) and 5% (IQR: -2% – +7%; data missing: 73 (62%)), respectively. The median values of T recorded inside the jet stall values varied minimally from take-off to landing. In particular, differences in T and H between one hour before landing and take-off were 1.0 °C (IQR: -2.0 – +6.0 °C; data missing: 49 (42%)) and 3% (IQR: -25% – +7%; data missing: 71 (60%)), respectively. The difference in T between arrival and departure varied, ranging from − 17.0 °C to 28.0 °C, with a median of 9.0 °C and an interquartile range of -1.4 °C to + 19.9 °C.

### Effects of air transport on horse health and behavior

#### Descriptive statistics of clinical signs and behavior and their association with the phases of air transport

At departure, most horses had an optimal BCS (i.e., BCS = 3/5) [16], which did not change between the transport phases monitored.

Descriptive statistics for the most commonly reported abnormal clinical signs and their possible association with the phases of air transport are shown in Table 4. The most common abnormal clinical signs were nasal discharge, increased capillary refill time (CRT) and abnormal demeanor recorded one hour before landing. Nasal discharge was most often bilateral and watery (Table S4, Additional File 1). A non-responsive or quiet demeanor was detected most often one hour before landing, whereas most horses were judged as bright at the departure airport. The color of the oral mucous membranes was most often normal (i.e., pale pink to pink), but two horses had red mucous membranes one hour before landing (Table S4, Additional File 1). Cough, abnormal defecation, and abnormal gut sounds were rarely detected. Two horses were coughing one hour before landing, and less than ten horses had altered gut sounds or defecation on arrival. Consequently, the presence of at least one abnormal clinical sign (see Table S2, Additional File 1) was associated with the air transport phases (*P* < 0.001), with a higher occurrence observed one hour before landing. Considering all transport phases together, 76% of horses (90/118 horses), including those with a history of TRPBs and TRHPs, showed at least one abnormal clinical sign (see Table S2, Additional File 1). Most were of no further clinical consequence.

**Table 4:** Number and percentage of abnormal clinical signs and their association with the different air transport phases

Variable	At departure (n (%))	One hour before landing (n (%))	On arrival (n (%))	One day after arrival (n (%))	Five days after arrival (n (%))	*P* value
Nasal discharge	12 (11)^A^	29 (28)^B^	13 (19)^AB^	0 (0)^C^	0 (0)^C^	**< 0.001**
Increased capillary refill time (CRT > 2 s)	28 (33)^A^	32 (34)^A^	nr	0 (0)^B^	0 (0)^B^	**< 0.001**
Abnormal rectal temperature (RT > 38.6 °C)	0 (0)^A^	nr	6 (9)	10 (13)^B^	11 (16)^B^	**0.001**
Presence of at least one abnormal clinical sign	41 (38)^ac^	56 (55)^Bbc^	30 (44)^c^	15 (20)^Aa^	20 (29)^A^	**< 0.001**
Abnormal demeanor	40 (37)^AC^	66 (65)^Bb^	18 (26)^Cc^	37 (49)^ABb^	29 (41)^ABac^	**< 0.001**

Total percentages: It is important to note that total percentages may not always sum to 100% for every characteristic due to rounding. nr: Not recorded in the survey. ns: Non-significant. Data were missing for nasal discharge at departure (missing = 11 horses, 9%), one hour before landing (missing = 16 horses, 14%), on arrival (missing = 50 horses, 42%), one day after arrival (missing = 43 horses, 42%), five days after arrival (missing = 48 horses, 41%); for increased capillary refill time at departure (missing = 32 horses, 27%), one hour before landing (missing = 24 horses, 20%), one day after arrival (missing = 72 horses, 61%), five days after arrival (missing = 74 horses, 63%); for abnormal rectal temperature at departure (missing = 48 horses, 41%), on arrival (missing = 50 horses, 42%), one day after arrival (missing = 43 horses, 36%), five days after arrival (missing = 48 horses, 41%); for abnormal demeanor at departure (missing = 9 horses, 8%), one hour before landing (missing = 16 horses, 14%), on arrival (missing = 50 horses, 42%), one day after arrival (missing = 43 horses, 36%), five days after arrival (missing = 48 horses, 41%). *P* value = effect of air transport phase. Values with different superscripts differ significantly (A, B, C = *P* < 0.001; a, b, c = *P* < 0.05)

The descriptive statistics of the numerical variables of heart rate (HR), respiratory rate (RR), and horse rectal temperature (RT) and their association with the air transport phases are shown in Table S5 of Additional File 1. The HR and RR changed significantly in relation to the air transport phase (*P* = 0.007 and *P* = 0.013, respectively), peaking at the departure airport and on arrival at quarantine facilities.

Horse behavior was affected by the different air transport phases. Pawing and head tossing were observed more often at the departure airport. Horse interaction was observed more at one hour before landing, whereas vocalizing, stamping, and turning the head were displayed more frequently on arrival. Licking or chewing was more often displayed one day after arrival, while resting was the predominant behaviors observed five days after arrival. Eating and sniffing were less often shown at departure (Table 5).

**Table 5:** Behaviors shown by the horses during one minute of scan sampling and their association with the different air transport phases

Variable	At departure (n (%))	One hour before landing (n (%))	On arrival (n (%))	One day after arrival (n (%))	Five days after arrival (n (%))	*P* value
Pawing	20 (19)^A^	8 (8)^a^	7 (10)	2 (3)^B^	0 (0)^Bb^	**< 0.001**
Tail swishing	5 (5)	5 (5)	6 (9)	8 (11)	1 (1)	0.073
Head tossing	25 (24)^A^	12 (12)	12 (18)^a^	10 (14)^a^	1 (1)^Bb^	**0.003**
Weaving	1 (1)	0 (0)	2 (3)	0 (0)	0 (0)	na
Vocalizing	11 (11)^A^	10 (10)^A^	21 (31)^B^	8 (11)^ACa^	0 (0)^Cb^	**< 0.001**
Stamping	6 (6)	5 (5)	7 (10)^A^	2 (3)	0 (0)^B^	**< 0.001**
Licking/chewing	19 (18)^A^	17 (17)^A^	17 (25)^a^	31 (42)^Bb^	24 (34)	**< 0.001**
Turning head	20 (19)^ADa^	11 (11)^AC^	35 (52)^B^	26 (36)^BDb^	20 (29)^BD^	**< 0.001**
Groom interaction	52 (50)	46 (45)	44 (66)	42 (57)	38 (54)	0.173
Horse interaction	35 (34)	50 (49)^A^	30 (45)^ACa^	19 (26)^B^	18 (26)^BCb^	**< 0.001**
Resting	66 (64)^A^	70 (69)^A^	39 (58)^A^	49 (67)^A^	66 (94)^B^	**< 0.001**
Sniffing	18 (17)^A^	18 (18)^A^	39 (58)^B^	35 (48)^B^	38 (54)^B^	**< 0.001**
Eating	12 (12)^A^	41 (40)^B^	40 (60)^Ba^	44 (60)^Bb^	40 (57)^B^	**< 0.001**

Total percentages: It is important to note that total percentages may not always sum to 100% for every characteristic due to rounding. na: Not applicable due to the low number of events. ns: Non-significant. For all the behaviors data were missing at departure (missing = 15 horses; 13%), one hour before landing (missing = 16 horses, 14%), on arrival (missing = 51 horses, 43%), one day after arrival (missing = 45 horses, 38%), five days after arrival (missing = 48 horses, 41%). *P* value = effect of air transport phase. Values with different superscripts differ significantly (A, B, C, D = *P* < 0.001; a, b = *P* < 0.05)

### Univariable and multivariable regression analysis between nasal discharge and increased CRT recorded one hour before landing and horse and journey details and air transport practices

Among the independent variables (see Table S6, Additional File 1) tested with nasal discharge as the outcome, those shown in Table 6 returned a *P* value < 0.100. When horses traveled in winter, they were more likely to develop nasal discharge than when they were transported during summer. Good-tempered horses were less likely to develop nasal discharge than bad and unknown-tempered ones, while feeding haylage increased the likelihood of nasal discharge being observed by three times in comparison with feeding grass hay. A decrease in the external temperature at the departure airport and in the number of horses transported in the cargo hold also influenced the reporting of nasal discharge.

The variables retained in the final multivariable logistic regression model for the observation of nasal discharge one hour before landing (model *P* value < 0.001) are shown in Table 6. Bad and unknown-tempered horses were more likely to develop nasal discharge particularly when they were moved in winter and autumn.

**Table 6:** Univariable and multivariable logistic regression models for the outcome variable of nasal discharge one hour before landing

Independent variable	Category	Beta (SE)	OR (95% CI)	Wald test *P* value	Log-likelihood test *P* value
**Univariable logistic regression models**					
**Air journey details**					
Journey season	Summer		Ref		**0.021**
	Autumn	1.8 (0.9)	6.1 (1.0-38.4)	0.053	
	Spring	0.5 (1.0)	1.7 (0.2–12.5)	0.620	
	Winter	2.1 (0.9)	8.5 (1.6–46.0)	**0.013**	
N° of horses in the cargo	-	-0.1 (0.0)	0.9 (0.9-1.0)	**0.010**	**0.008**
**Horse details**					
Horse temperament profile	Good		Ref		**<0.001**
	Bad	1.7 (0.6)	5.3 (1.5–18.4)	**0.009**	
	Unknown	2.2 (0.6)	9.3 (2.9–29.4)	**< 0.001**	
**Air transport practices**					
Jet stall location	At extremities		Ref		0.099
	In between wings	0.8 (0.4)	2.2 (0.8–5.8)	0.102	
Food type	Grass hay		Ref		**0.032**
	Haylage	1.2 (0.5)	3.3 (1.1–10.1)	**0.034**	
External environmental temperature at departure airport (°C)	-	-0.6 × 10^− 1^ (0.0)	0.9 (0.9-1.0)	**0.003**	**0.002**
**Multivariable logistic regression model**					
Journey season	Summer		Ref		**0.024**
	Winter	1.2 (0.8)	7.4 (1.5–36.4)	**0.015**	
	Spring	1.5 (1.0)	4.5 (0.6–34.2)	0.148	
	Autumn	2.7 (1.0)	14.7 (2.2–98.1)	**0.006**	
Horse temperament profile	Good		Ref		**<0.001**
	Bad	1.7 (0.6)	5.5 (1.6–18.6)	**0.007**	
	Unknown	2.5 (0.7)	12.5 (3.1–50.4)	**< 0.001**	

Significant *P* values (i.e., *P* value < 0.05) are shown in bold

Among the factors (see Table S7, Additional File 1) evaluated with abnormal capillary refilling time as the outcome, those shown in Table 7 returned a *P* value < 0.100. Horses subjected to stops *en route* were less likely to be assessed with an abnormal CRT compared with horses on direct flights. Similarly, during air journeys of shorter duration, and those which did not require quarantine (N.B. these often were the same journey from Europe to the USA), horses were more likely to have an abnormal CRT. In addition, an increase in the number of horses transported in the cargo holds increased the likelihood of having horses with an abnormal CRT. Finally, horses with the head tied short were eight times more likely to display increased CRT than horses tied long or untied.

The variables retained in the final multivariable logistic regression model for the observation of increased CRT one hour before landing (model *P* value < 0.001) are shown in Table 7. Horses with unknown experience in road travel and traveling a shorter distance were more likely to develop increased CRT one hour before landing.

**Table 7:** Univariable and multivariable logistic regression models for the outcome variable of increased capillary refill time one hour before landing

Independent variable	Category	Beta (SE)	OR (95% CI)	Wald test *P* value	Log-likelihood test *P* value
**Univariable logistic regression models**					
**Air journey details**					
Stops	Yes		Ref		**< 0.001**
	No	2.8 (0.8)	16.0 (3.3–78.2)	**< 0.001**	
N° of horses in the cargo	-	0.1 (0.0)	1.1 (1.0-1.1)	**0.015**	**0.013**
Flight duration (h)	-	-0.4 (0.1)	0.7 (0.6–0.8)	**< 0.001**	**< 0.001**
**Horse details**					
Horse experience in road travel	Travel regularly		Ref		0.083
	Few trips	-0.1(0.7)	0.8 (0.2–3.5)	0.829	
	Unknown	1.0 (0.6)	2.8 (0.8–9.5)	0.087	
**Air transport practices**					
Quarantine	Yes		Ref		**<0.001**
	No	2.9 (0.6)	17.8 (5.9–53.9)	**< 0.001**	
Horse tying	Tied long or untied		Ref		**<0.001**
	Tied short	2.2 (0.5)	8.7 (2.9–25.6)	**< 0.001**	
**Multivariable logistic regression model**					
Flight duration (h)	-	-0.4 (0.1)	0.7 (0.6–0.8)	**< 0.001**	**< 0.001**
Horse experience in road travel	Travel regularly		Ref		**0.043**
	Few trips	0.9 (0.7)	2.4 (0.6–9.8)	0.226	
	Unknown	1.5 (0.6)	4.3 (1.3–13.7)	**0.014**	

Significant *P* values (i.e., *P* value < 0.05) are shown in bold

### Incidence of air transport-related injuries and health problems (TRHPs)

None of the transported horses died but 22 (18.6%) developed TRHPs. Ten horses reported at least one injury during the transport phases monitored. One horse was injured at a quarantine facility and six were found injured at the departure airport, including six shallow cuts, one deep cut and one with swelling. Injury locations were forelimb in five horses, head in three and hindlimb in one. One more horse developed a swelling on the chest during the air journey. On arrival at quarantine, one horse had a shallow cut on the hindlimb. Another was injured during the quarantine period, with skin abrasion on the head. Most of the transport-related injuries were due to transport by road or quarantine and happened to horses with an unknown travel history.

After the journey, two horses had diarrhea without pyrexia; one of these was the horse who had a swollen chest. A total of 11 horses (11/118; 9%) developed pleuropneumonia (i.e., shipping fever). Among these 11 horses, two were reported to have a history of TRPBs and a transport-related injury, and six had an unknown travel history. These horses traveled in two separate shipments, shipments 23 and 29. These shipments involved small groups of horses (6 and 12 horses, respectively), which were transported over the longest distances by road from quarantine to the departure airport (600 and 697 km, respectively), and moved long distances from one hemisphere to the other (i.e., from the USA to New Zealand and Australia, respectively) and therefore transiting opposite seasons and environmental conditions (i.e., air temperature and relative humidity) between departure and arrival. These shipments involved different breeds of horses stabled in jet stalls on pellet-based bedding. All horses were immediately treated following diagnosis, and the agents informed us that they recovered.

## Discussion

The study followed over 100 horses, documenting their journeys, key practices associated with their international transport by air, behavioral and health challenges to their welfare, and associations between practices and some welfare issues. As hypothesized, the air transport affected the behavior and health status of the monitored horses, with stress-related behaviors (e.g., pawing, head tossing) shown more at the beginning of the journeys, and abnormal clinical signs and tiredness (i.e., horses not alert, all very calm and resting more with the head down) more evident before landing. In addition, details of the horse (e.g., temperament) and journey (e.g., stops) and air transport practices (e.g., tying the horse) were associated with the manifestation of some clinical signs, supporting our hypothesis.

The numerically representative target of 106 horses was exceeded, and although numerically representative, it is unlikely to be demographically so. Although it has been estimated that approximately 10,000/year horses travel by air, little is known of that population’s characteristics [17]. A study of 869 horses transported by air to Hong Kong over two years was comprised of 98% male horses; 87% were Thoroughbred racehorses < 6 years old, and the remainder were riding ponies up to 18 years old [11]. Oertly et al. [14] followed a group of 122 competition horses flown on an international competition circuit of whom 34% were mares, 23% were stallions, and 43% were geldings with a median age of 11 years. In contrast, the current study included a wider scope of equestrian activities and breeds, with Warmbloods and Thoroughbreds predominating. Furthermore, this population was predominantly female and had a median age of 7 years. Of interest, a quarter of the transported mares were pregnant, and over 10% of horses were between eight months and two years old. Age status should be considered carefully. Foals are at a greater risk of injury in road transport [18], but the welfare risks to them in air cargo stalls have not been described. Pregnancy status should also be carefully considered for any type of transport [5, 19] because this may predispose broodmares to stress [20], alter parturition [21] or induce illness [22]. For pregnant mares and young horses, it is compulsory to provide more space during road transportation [19], but in our study, industry standard-sized three-horse jet stalls were used. In addition, potentially unbroken young horses were transported over long distances (> 8 h), contrary to what is indicated for this category for road transport in the European Regulation [19]. There are no definitive studies on the effects of air transport on pregnant mares and their foals or unbroken horses, or specific regulations for the air transport of these categories of horses. For the moment, the authors suggest that pregnant mares should be transported in two-horse jet stalls to meet the recently published recommendations on minimal space allowance during road transport published by EFSA [5].

In the current study, most shipping agents did not know the travel history of the horses they were moving. Many horses with an unknown travel history developed injuries and TRHPs, including shipping fever. Knowing the travel history of a horse transported by air is fundamentally important for transport management because negative travel-related experiences may predispose them to the development of TRPBs and compromised welfare [5, 13, 23]. Whether horses with TRPBs could be pharmacologically supported before air transport has not been proven yet [5]. Moreover, in this study population, a proportion of horses had little experience in road travel, and most had not received any related training. Lack of experience and training to travel are risk factors for TRPBs and injuries in horses and horse handlers [23, 24]. Considering that air transport is a complex event, consisting of several ground and air phases [4, 8], it is, therefore, advisable that horses should be well experienced and trained for transport and preferably without previous experience of TRPBs and TRHPs before being subjected to air transportation [5] to reduce negative outcomes for handlers and horses. Shipping agents should ask owners or consignors about travel history and manage horses accordingly.

In this investigation, all the horses were transported by road to the quarantine facilities and the departure airports. Road transport of horses is widely recognized as a welfare concern, as it can predispose horses to the development of various TRPBs and TRHPs [2]. It is therefore advisable to minimize road transport before the air journey (e.g., always choosing the closest quarantine to the airport) to reduce the risks associated with it. Once at the departure airport, all horses included in the study waited multiple hours in the road vehicles and the jet stalls before being loaded into the air cargo holds. The air temperature and relative humidity can increase rapidly in stationary vehicles (or stationary jet stalls) [5] exposing horses to the risk of thermal stress [5]. Peak air temperatures and relative humidity were recorded inside the jet stalls after remaining stationary for multiple hours at the departure airport during summer months. The authors and other researchers advise keeping waiting times to a minimum [13]. In cases where delays cannot be avoided, it is recommended not to expose vehicles or jet stalls directly to the sun or extremely cold conditions [5]. When air temperature is above the thermal comfort zone of the horses, fresh water and an effective ventilation system should be provided to support thermoregulation [5, 13].

During the flights monitored, the environmental conditions inside the jet stalls remained in a steady state, as expected [10]. Although the air temperature and relative humidity values were higher in the jet stall than in the cargo hold during the flight, the artificially controlled conditions in the cargo bay maintained the jet stall within the thermoneutral zone of the horses [5]. However, in the current study, ten journeys out of 32 (31%) had one or more *en route* stops, and more than half of the monitored population was subjected to stationary periods during the flight. During stops, the air temperature in the cargo holds cannot be artificially controlled and conditions (i.e., air temperature and relative humidity) are influenced by the external ambient conditions [10] predisposing animals to respiratory disorders [25]. It is therefore advisable to perform stops *en route* carefully [13]. Ideally, stops should be scheduled specifically for the horses and not for other logistical reasons such as loading additional goods. Overall, planning the air journey carefully is essential to enhance horse welfare. Other than air temperature fluctuations, horses in the jet stalls were exposed to poor lighting. Light was unfortunately not objectively measured but it is worth highlighting that there are no specific requirements for lighting in equine air transport. It could be advisable to add a short wavelength blue light [26] in the jet stall, as the maintenance of a light-dark cycle is essential to respect circadian rhythms and the welfare of animals [27]. Moreover, light is crucial to monitor the horses, and bright light sources may annoy horses [28]. More studies on the optimal type of light to provide in the cargo hold and the jet stalls are recommended.

Associations between physiological and behavioral parameters and the air journey phase were found. Horse interaction was significantly associated with the stationary phase of the flight, whereas stress behaviors and increased heart and respiratory rates were mostly associated with transitional events, such as departure and arrival. This was expected and in agreement with the literature as it has been reported that horses tend to be more relaxed during the flight, and more agitated during take-off and landing [3, 8, 10]. Horses may eat and drink less during transport than they would in their normal environment due to stressful and unfamiliar circumstances [2, 8]. Others have observed that during flight, horses are relaxed enough to eat regularly [8]. In the current population investigated, almost all horses ate and drank during the flight, but many initially did not accept water. This hesitation to drink may be due to the short time window given to the horse to adapt to an unfamiliar and uncomfortable position, or the inflight sequence of feeding and watering, as many horses drink only after eating [5, 29]. This study is the first to report the average feed consumption and water intake, which were in line with their daily requirements [30]. These data demonstrate the importance of offering feed and water as often as possible during the journeys [5].

A non-responsive or quiet demeanor was observed more frequently one hour before landing. This should not be interpreted as a sign of relaxation, and in the circumstances studied, it could be construed as a sign of fatigue due to journey duration. The total journey duration starting from the quarantine, including waiting time and flight duration when often many hours were spent in limited spaces without the opportunity to stretch or lie down, was indeed always quite long (more than 12 h). Signs of fatigue, measured by a quiet and non-responsive demeanor and increased blood levels of lactate and muscle enzymes, were found in horses transported by road for 8 h [32]. Air transport has been described by other authors [3] to be less stressful than other types of transport for horses. However, the authors of the current work suggest it should continue to be considered a welfare hazard, as it may expose horses to the risk of welfare issues. During the current study, signs of distress during transitional phases and at least one abnormal clinical sign were indeed detected in almost all horses. Monitoring the physiological parameters and behavior of each horse during each phase of the air journey could be crucial in minimizing the risk, allowing early identification of poor welfare. However, flight grooms have limited time to access the cargo hold for safety reasons (i.e., the flight groom is untethered in the event of turbulence) and micro-environmental reasons (i.e., pressure surges in the cockpit), and the number of flight grooms is limited by the number of seats available on the aircraft. Thus, innovative technologies that remotely monitor horse physiology and behavior during the air journey should be implemented to facilitate the flight grooms’ jobs and enhance horse welfare.

In addition to changes in physiological and behavioral parameters, nasal discharge and increased CRT were influenced by the air transport phase, being more frequent one hour before landing. Nasal discharge is a clinical respiratory sign. In the univariable logistic regression models, its manifestation was increased by lower external temperatures and the coldest season, in agreement with findings for road transport [2, 5]. Accordingly, thermal stress during air journeys should be minimized, as indicated for other types of live animal transportation [25]. The univariable model associations between nasal discharge and the other factors, namely bad temperament, food type, and number of horses in the cargo hold, are novel findings. These factors may have impaired the respiratory clearance of horses and have been impacted by the microclimate of the jet stall. In the multivariable logistic regression model, only season and temperament remained significantly associated with nasal discharge as an outcome. These final findings were expected and confirm our hypothesis. Temperament is a genetic trait [31], and horses with a poor temperament can have difficulty coping with stressors, tend to be more alert, and therefore maintain a high neck position, which impairs respiratory clearance [32]. More studies on the effects of genetics on behavior and welfare are advisable to enhance animal welfare during travel [33].

An impaired CRT is a clinical sign of compromised tissue perfusion associated with systemic illness or dehydration. It is therefore not surprising that in the univariable logistic regression models, factors that may impede watering, such as tying the horse too short during air travel or performing flights without stops increase the likelihood of having horses with increased CRT before landing. Leadon [13] has suggested that stops could be beneficial for horses by giving them more time to drink in quieter conditions. It is therefore be advisable to allow horses to drink freely, leaving them untied or tied on a long lead during the journeys and stops so that they can move unhindered. This would facilitate comfortable water access to reduce dehydration and head movement for airway clearance, reducing the occurrence of nasal discharge and the risk of respiratory disorders [32]. In addition, it is always recommended to monitor the horses’ water intake during the journey. In the multivariable logistic regression model for CRT, only journey duration and experience in road travel remained significant. The negative association with flight duration may appear contradictory. However, in the current study, horses moved on short flights are unlikely to have sufficient time to be provided with water and drink to replace losses sustained during the pre-flight phases of the journey (e.g., waiting in the jet stall on the tarmac). Grooms offer water in a rationed manner in a bucket for a short time and then quickly move on if the horse does not drink. They have to water all horses in the cargo hold within a limited window of time and know that many horses do not drink within the first 6–7 h of flight (grooms’ personal communications to BP). This is more challenging when there are more horses in the cargo hold, increasing the likelihood of one or more horses with signs of dehydration. The association in the multivariable logistic regression model between the increased CRT and the unknown experience of travel reinforces the importance of noting the horse’s history. The authors suggest training horses for travel, as they may be less scared and start drinking earlier. Considering that the journey of most horses begins with a period of quarantine and the long waits recorded at the departure airports in trucks and jet stalls, it is suggested water be offered in each of these locations. This practice was rarely recorded during this study.

In the current study, a fifth of the horses developed an injury or TRHPs. In agreement with the literature, injuries were recorded most often associated with road transport [24] and quarantine. Quarantine is necessary to prevent the spread of infectious diseases, but nevertheless, it represents a welfare hazard [2, 4]. The novel environment of the quarantine facility can expose horses to restraint-related and social stress [2, 9] that may lead to the reactivation of latent viral infections [34] or injury, as observed in the current case. It is therefore advisable to review quarantine management practices, individually tailoring them to minimize the impacts of quarantine stress on equine welfare. Road transport towards the departure and from arrival airports should be minimized to that which is necessary to reduce the effects of this transport phase on equine health and welfare. In addition to injuries, cases of shipping fever were detected, but the incidence was lower (9%) compared to other studies monitoring horses traveling by air over long distances [11, 14]. The difference may be due to an increased awareness of the factors that can cause the disease following the publication of earlier studies, and early detection and treatment. However, it is worth noting that the incidence found in the current study could be underestimated as we did not include other diagnostic tests after arrival. Furthermore, RT was not monitored during the journeys, so transitory pyrexia during the flight, as reported in prolonged road transport, may not have been detected [35].

Overall, 76% of the horses developed an abnormal clinical sign during the journey. However, no horses died, and all of them recovered. This may be due to the applied transport practices performed by the experienced flight grooms, quarantine officers and veterinarians who managed these horses. The experience of flight grooms in handling and monitoring the horses closely may have prevented injuries during the ground phases at the airport and during the flight. The experience of flight grooms and veterinarians in recognizing minimal clinical signs of shipping fever and their prompt intervention resulted in positive health outcomes. In general, all staff dealing with air transport should be adequately trained in horse behavior, health, handling, and management, as it has been reported that the experience of the handler is crucial to minimizing injuries and poor welfare during animal transportation [5, 13, 24].

Our findings need to be interpreted with caution since this study has several limitations. One limitation was that the total air journey duration from loading at the departure destination quarantine facility to unloading at the arrival location quarantine facility was not calculated. This data would provide a more accurate value for the duration of the journey encompassing all phases, but its calculation was hampered due to the multiple data blocks being completed by different people during each travel event. The measurement of some clinical signs, particularly rectal temperature and gut sounds, was only possible for some phases of air transport. This occurred because horses were confined to the jet stalls and difficult to monitor, and no other tools were available to monitor clinical parameters within them. Another challenge was that many variables could not be modeled to examine their influence on health and welfare outcomes due to the high number of missing data. The authors elected not to impute missing data due to the relatively modest sample size. Missing data was mainly due to the high number of people involved in the air transport practices, and the limited time available for the grooms to perform their duties and collect data. This could have been mitigated using video cameras or other wearable sensors on the horses, but they were not permitted because of aircraft operating procedures. It would be ideal for future studies to monitor horses using smart technologies [36] or have a person dedicated to the data collection during the flights. Information on management practices at the quarantine facilities of the destination countries was also missing. It is suggested that future studies include questions about the practices performed in the quarantine of the arrival countries. Data were only collected by four cooperating shipping companies and on a limited number of routes. This may have underestimated or overestimated some of the findings, which could be clustered for the routes monitored or the practices applied. The agent collecting the data was the same for each shipping company, but the grooms were different on some routes, so data were collected by different assessors. Even if assessors were trained in data collection, individual biases cannot be excluded. The models were not corrected by shipping company, air cargo company, or journey ID, as the dataset was too small, and the logistic regression models did not converge. However, in future studies with larger samples, it may be interesting to investigate whether different shipping companies and/or air cargo companies, based on their practices, have different health and welfare outcomes for the horses they transport. Information on the individual characteristics of some horses was lacking and the number of events per health problem was low. Therefore associations could not be tested in some cases. Finally, due to the lack of a validated protocol to assess the overall welfare of horses after traveling, it was not possible to analyze the multifaceted aspects of health and behavior as an outcome, and we focused our analysis on the most robust and reliable health-related data available. Notwithstanding these limitations, to the best of the authors’ knowledge, this is the first study investigating both the practices performed during equine air transport and the factors associated with air transport welfare issues. These data provide novel details about the air transport of horses and could be useful in developing evidence-based best practices to help industry stakeholders and policymakers to improve the welfare of horses transported by air. Our current findings also provide a valuable foundation for future studies.

## Conclusion

The current study contributes to our understanding of the air transportation of horses which was confirmed to be a complex event that can impair horse welfare. The impact of air transport on horse welfare depends on numerous factors, making each air journey a unique story for each horse. Despite the modest sample size, our data provide novel evidence from which the following recommendations can be suggested: (i) Shipping agents should always obtain the past travel history and the temperament of the horses to be shipped to adapt management practices to them; (ii) Unbroken horses and those with an extremely poor temperament should not be shipped by air. All horses should have been trained for shipping, and then handled by staff with knowledge of animal learning and behavior; (iii) During the flight, horses should not be tied short, and their water intake should be monitored and recorded; (iv) During very long journeys, during the stops, horses should be unloaded from the jet stalls and kept in appropriate facilities to be watered, fed, and have a rest. Logistic stops to fill the cargo with other pellets should be instead avoided; (v) The grooms should have increased time for access to the cargo, and monitoring of the horses, or the ratio between horses/grooms should be increased. Moreover, it would be useful to allow the use of cameras and novel technologies to remotely monitor clinical parameters, such as RT and RR, and alert the grooms in case of problems.

These recommendations need to be confirmed in future studies but may be useful to implement with the current IATA regulations and to motivate stakeholders to apply evidence-based best practices to protect horse welfare during transportation by air.

Only proper management applied by experienced staff can help horses cope with air transport with minimal impact on their health and welfare.

### Methods

#### Population

The studied population was client-owned horses transported internationally by commercial aircraft from 2020 to 2023. A power analysis was performed to determine the minimum sample size of horses to monitor in the study based on a population of 40,000 transported horses, estimated using data reported in the literature, namely approximately 10,000 horse movements per year [17]. The minimal sample size required was 106 horses. This sample size was determined by assuming an expected proportion of shipping fever of 11% [11], with 5% precision and a 90% confidence interval (CI). Non-equid species were excluded.

### Data collection

Data on horses were collected through a standard protocol, including seven blocks of questions, completed by those associated with separate phases of each equine transport event (Table S1, Additional File 1).

The first and the second blocks were completed by the shipping agents, who were always the same for each of the shipping companies (i.e., four shipping agents collected the data for the four shipping companies). The first block consisted of 14 closed and three open-ended questions asking about owners’/trainers’ backgrounds and expertise in the equine industry (e.g., sector of involvement), and their horses’ history of training for travel, previous transport-related problem behaviors (TRPBs), and transport-related health problems (TRHPs). The second block consisted of eight closed and nine open-ended questions on horses’ details (e.g., sex, age, breed) and how they were transported to the departure airport (e.g., commercial, or non-commercial transportation, type of vehicle). If horses were quarantined, five more closed-ended and three more open-ended questions were asked. The latter concerned the management of quarantined horses (e.g., quarantine duration, exercise type, and frequency during quarantine), and the distance of the quarantine facility from the departure airport. The block closed with two questions about the fitness for transport (i.e., if the horses were evaluated as fit for transport and who had assessed their fitness for travel) and three questions about flight number, airline name, and time of arrival at the airport.

The third block was completed by the flight grooms at the departure airport, consisting of 13 closed and 11 open-ended questions. This block collected information on the years of experience of the flight groom in handling horses, clinical signs (e.g., nasal discharge) and the behavior of the horse. The clinical signs (Q54-Q67 in Table S1, Additional File 1) were assessed using a visual clinical inspection, while a 1/0 sampling method ethogram (Q68 in Table S1, Additional File 1) was used for recording the occurrence of specific behavior during one minute of direct observation. The grooms judged how easily the horses loaded in the jet stall (Q71) and recorded information about the external environmental temperature at the departure airport (Q51) and the air temperature and relative humidity inside the stall at take-off (Q69, Q70) using weather stations.

The fourth block of questions addressed details about the air journey practices and was completed by the flight grooms one hour before landing. A total of 32 closed and 16 open-ended questions on horses’ clinical signs (Q91-102 in Table S1, Additional File 1), horse management practices (e.g., frequency and amount of feeding and watering), the horses’ behavior (same ethogram of previous block Q103 and a total behavioral score reported in Q110 of Table S1, Additional File 1), and journey details (e.g., numbers and locations of the stops, when performed, and unloading of the animals from the cargo or the jet stalls during the stops) were included in this block.

Questions in the last three blocks were completed by shipping agents and quarantine officers/veterinarians, and focused on the same clinical signs and behavior recorded previously, but included specific questions on possible health conditions affecting the horses (e.g., shipping fever) (Q146, Q167, Q190 of Table S1, Additional File 1). These last three blocks were filled immediately after, one and five days after arrival, respectively. At the beginning of the fifth block, further information about the external environmental conditions at the airport of arrival, the country of arrival, the time zone difference and the flight duration were collected. The fifth block consisted of 17 closed and ten open-ended questions, while the sixth and seventh blocks consisted of 17 closed and six open-ended questions. The standard protocol was developed by a process of iterative review by the researchers, piloted by one of the authors (BP) with 24 horses transported internationally by air, published using Qualtrics Software (Qualtrics, Provo, UT, USA), and promoted among Animal Transportation Association (ATA) members.

In 2020, one of the authors (BP) asked ATA [37] for the contact list of its members (*n* = 43) involved in the equine air transportation industry. Then, the researchers emailed these members, namely International Shipping Companies (ISCs) and airlines transporting equids, to explain the project and ask for data collection or sponsorship collaboration. With the four ISCs who agreed to collaborate, the researchers organized several meetings to explain how to use the developed protocols. In particular, the researchers trained these four shipping agents and various flight grooms to collect data and upload them online. Moreover, one of the researchers (BP) personally participated in some air journeys to refine the training and collect some data in person. Data collection was performed from 2020 to 2023 on subgroups of horses randomly chosen by the shipping agents belonging to larger groups that traveled internationally by air. For example, if 14 horses were being transported, the agent would randomly select six to monitor more closely.

### Data handling and definition of the variables

The data were organized in Microsoft Excel® (Microsoft® Excel® for Microsoft 365 MSO Version 2306 Build 16.0.16529.20100). In the Excel sheet, each row represented a horse, and each column contained the answer to each question (i.e., a variable). Names, definitions, and data handling of all the categorical and numerical variables subjected to descriptive or further statistical analysis are shown in Tables S2 and S3 of Additional File 1.

### Statistical analysis

Data of the retained variables (Tables S2 and S3, Additional File 1) were used to generate descriptive statistics and results are shown as the minimum (min), maximum (max), median and interquartile range (IQR) for each of the numerical variables and as counts and percentages for each of the categorical variables. Clinical and behavioral binary responses (i.e., presence/absence of nasal discharge, increased CRT, abnormal RT, abnormal demeanor, presence of at least one abnormal clinical sign, and the occurrence/non occurrence of pawing, tail swishing, head tossing, weaving, vocalizing, stamping, licking/chewing, turning head, groom interaction, horse interaction, resting, sniffing, eating) were analyzed by Generalized Estimating Equations procedures with binomial as probability distribution and logit as link function, horse ID as subject factor and the air transport phases (i.e., at departure airport/one hour before landing/on arrival/one day after arrival/five days after arrival) as within-subject factor. Unstructured covariance matrix for repeated effects was used. Finally, Sidak was used for multiple comparisons. The Friedman Rank Sum Test was used to explore associations between the air journey phases and the numerical clinical outcomes (i.e., HR, RR and RT).

Univariable and multivariable logistic regression models were performed to separately examine the associations between a range of independent variables (related to air journey and horse details, and air transport practices) and (i) nasal discharge (present/absent) and (ii) increased capillary refill time (CRT; present/absent) recorded one hour before landing. The full list of the independent variables is shown in Table S8 of Additional File 1. Among all variables (Tables S2 and S3, Additional File 1), those with less than or equal to 10% missing values and with balanced categories (> 5%) were included in the list. In addition, if a variable was expressed as either categorical or numerical, only one variable format was included in the univariable and multivariable logistic regression models.

For the univariable analysis, separate unmatched logistic regression models were used to determine the association between each health variable (i.e., outcome) and the independent variables and significance was assessed using the likelihood ratio test and Wald test. Among the full list tested (Table S8, Additional File 1) several independent variables were excluded for a specific outcome because the model would not converge; Table S6 and S7 of Additional File  1 show the Wald test *P* values of the variable tested for the outcome of nasal discharge and increased CRT, respectively. In the analysis of each outcome, variables that were associated with the outcome at a significance level of *P* < 0.100 were initially selected for potential inclusion in the multivariable logistic regression model. However, to address concerns of collinearity, where variables represented closely related factors, careful consideration was given to which variables to include. For instance, in the multivariable logistic regression model examining the occurrence of nasal discharge, we faced a choice between including either “Journey season” or “External environmental temperature at departure airport” as variables. Ultimately, “Journey season” was chosen for inclusion. Similarly, in the increased CRT (Capillary Refill Time) multivariable logistic regression model, variables such as “Stops”, “Quarantine”, and “Flight duration” were associated. Of these, “Flight duration” was retained for the final model. Once the preliminary list of variables for inclusion was established, we employed a manual backward elimination process to refine the model. This method involved systematically removing the least significant variable, continuing until all remaining variables demonstrated an association with the outcome at a significance level of *P* < 0.05. The significance of variables was assessed using both the likelihood ratio test and the Wald test statistic. Descriptive statistical analyses were performed using R software (R Version 4.2.3; www.r-project.org) [38]. All the univariable and multivariable logistic regression models were performed using Gen Stat® Version 21.1 (VSNInternational, Hemel Hempstead, UK) with an estimate of lack of fit as an option.

### Electronic supplementary material

Below is the link to the electronic supplementary material.

Additional File 1

Additional File 2

Additional File 3

Additional File 4

## Data Availability

The dataset used and/or analysed during the current study is available from the corresponding author on reasonable request.

## Abbreviations

IATA: International Air Transport Association
ATA: Animal Transportation Association
EFSA: European Food Safety Authority
TRPBs: Transport-Related Problem Behaviors
TRHPs: Transport-Related Health Problems
T: Temperature
H: Humidity
IQR: Interquartile Range
min: Minimum
max: Maximum
kg: Kilograms
km: Kilometers
°C: Degree Celsius
h: Hour(s)
L: Liters
BCS: Body Condition Score
CRT: Capillary Refill Time
RT: Rectal Temperature
HR: Heart Rate
RR: Respiratory Rate
EC 1/2005: Council Regulation (EC) No 1/2005 of 22 December 2004
ISCs: International Shipping Companies
