# Supplementary material for: Analysis of current methods and Welfare concerns in the transport of 118 horses by commercial air cargo companies
Source: BMC Vet Res. 2024 Apr 26;20:158. doi: 10.1186/s12917-024-03999-9 (PMC11046973; doi:10.1186/s12917-024-03999-9)
Supplement: Supplementary file 1 — Additional File 1 [file 12917_2024_3999_MOESM1_ESM.docx]

**Supplementary tables**

**Table S1.** The standard protocol used for the data collection on a sample of 118 horses from 597 horses transported internationally by air from 2020 to 2023.

| **Default Questions Block** | |
| --- | --- |
| Name of the horse (as reported on the passport) ____________ | |
| Which of the options below describes your role?   - Operator asking questions to the owner or authorized agent (i.e., trainer) before departure - Shipping agent of the horse before departure/quarantine manager - Flight groom or veterinarian at the departure airport - Flight groom or veterinarian at the end of the journey (1 hour before landing at destination) - Flight/receiving groom/quarantine manager/veterinarian at arrival quarantine/destination stall - Quarantine officer/veterinarian or owner/agent one day after the journey - Quarantine officer/veterinarian or owner/agent five days after the journey | |
| **BLOCK 1 - Questions for the owner or authorized agent (i.e., trainer)** | |
| Q1. Which sector of the horse industry are you primarily involved in?   - Thoroughbred racing - Standard racing - Breeding - Showjumping - Dressage - Eventing - Showing - Endurance - Western sport and performance (e.g., reining, barrel) - Pony Club - Recreational riding - I don’t know - Other (please specify) ____________ | |
| Q2. (*If it was answered "Breeding" to Q1*) What horse breed do you breed?   - Thoroughbred - Standardbred - Sport horses - Other (please specify) ____________ | |
| Q3. How many years of experience does the owner/trainer have with handling horses? ____________ | |
| Q4. What is the level of involvement of the owner/trainer in the equine industry?   - Professional (e.g., involved with horses for work/financial gain) - Amateur (e.g., involved with horses as a hobby) - I don’t know - Other (please specify) ____________ | |
| Q5. In the busiest week of the year, approximately how many horses are on your property or place of equine business together with the shipped horse?   - < 5 horses - 5-10 horses - 11-30 horses - 31-50 horses - 51-100 horses - More than 100 horses - I don’t know | |
| Q6. And how many are now on the property of business premises? ____________ | |
| Q7. What type of the following training for loading/traveling has the shipped horse been subjected to?   - No specific transport-related training - The horse was already trained by an unknown training method - Self-loading (i.e., the horse loads at visual or auditory command by himself) - Habituation training (i.e., the horse was exposed to loading, traveling and unloading procedures before the real travel) - Positive reinforcement and negative punishment (e.g., clicker training) - Negative reinforcement and positive punishment (e.g., dressage whip, whip, bum rope etc.) - I don’t know - Other (please specify) ____________ | |
| Q8. Did the horse show behavioral problems associated with loading, unloading or during shipping in the past?   - Yes - No - I don’t know | |
| Q9. (*If it was answered "Yes" to Q8*) What type of behavioral problems?   - Anxiety - Freezing - Kicking - Scrambling - Rearing - Biting - Refusal to load - Refusal to unload - Escape behavior - I don’t know - Other (please specify) ____________ | |
| Q10. (*If it was answered "Yes" to Q8*) In which phase/s of transport did the horse show behavioral problems?   - Preloading (e.g., anxiety, pawing) - Loading (e.g., refusing to load) - Traveling (e.g., scrambling, kicking, biting) - Unloading (e.g., refusing to load, jumping) - Post-travel (e.g., anxiety, refusing to eat, vocalization) - I don’t know | |
| Q11. What is the horse's experience with road traveling before this journey?   - First trip - Has had a few trips - Travel regularly - usually calmy and without problems - Travel regularly - usually anxious or uncomfortable or poorly behaved - I don’t know | |
| Q12. Please grade (from never to most of the time) the temperament of this horse | |
| Playfulness  Friendliness  Nervousness/Excitability  Stubbornness  Attachment to horse(s)  Understanding | ○ Never ○ Sometimes ○ Most of the time ○ I don’t know  ○ Never ○ Sometimes ○ Most of the time ○ I don’t know  ○ Never ○ Sometimes ○ Most of the time ○ I don’t know  ○ Never ○ Sometimes ○ Most of the time ○ I don’t know  ○ Never ○ Sometimes ○ Most of the time ○ I don’t know  ○ Never ○ Sometimes ○ Most of the time ○ I don’t know |
| Q13. How do you judge the horse in loading into a vehicle?   - - Easy to load, minimal assistance required   - Moderately resistant to load, moderate assistance required   - Resistant to load, forcefully assistance required - Highly resistant to load, maximal assistance or sedation required   - Never loaded before - I don't know | |
| Q14. Has the horse experienced any transport-related diseases or injuries in the previous two years?   - - Yes   - No - I don’t know | |
| Q15. (*If it was answered "Yes" to Q14*) What type of transport-related diseases or injuries has the horse experienced in the previous two years?   - Shipping fever (equine transport pneumonia) - Colic - Heatstroke - Diarrhea - Injury - Poor appetite - Increased temperature - Muscle injury or bruise - Fracture - Head injury - Other (please specify) ____________ | |
| Q16. (*If it was answered "Yes" to Q14*) Please give more details on the incident(s) (i.e., the type of journey, when it happened, the outcome etc.) ____________ | |
| Q 17. Did the horse receive any treatment within the week before undertaking the journey from the home stable to the airport/quarantine?   - Administration of antibiotics - Administration of tranquilizers - Administration of vaccinations - Administration of intravenous fluids - Administration of oral supplements (e.g., electrolytes, vitamins) - Removal of shoes - Application of bandages/travel boots - Wearing of rug - No treatment - I don't know - Other (please specify) ____________ | |
| **BLOCK 2 – Questions for the shipping agent of the horse/quarantine manager before departure** | |
| Q18. Name of the company of the shipping agent ____________ | |
| Q19. What sex is the horse?   - - Stallion   - Gelding   - Mare - Pregnant mare | |
| Q20. How old is the horse? (In years, please) ____________ | |
| Q21. What breed is the horse?   - Thoroughbred   - Standardbred   - Warmblood   - Arab   - Quarter horse   - Draft horses   - Pony - Other (please specify) ____________ | |
| Q22. Height of the horse (in cm, please) ____________ | |
| Q23. Body weight of the horse (in kg, please) | |
| Q24. Reason for moving the horse   - - Racing   - Equestrian competition   - Sale   - Breeding   - Moving as part of personal relocation - Other (please specify) ____________ | |
| Q25. Stable of origin (City) ____________ | |
| Q26. Stable of origin (Country) ____________ | |
| Q27. Name of the departure airport ____________ | |
| Q28. Name of the country of the departure airport ____________ | |
| Q29. How long did the horse travel to reach the airport? (Distance in km, please) | |
| Q30. How did the horse reach the airport?   - - Transport company - Non-commercial transportation | |
| Q31. (*If it was answered "Transport company" to Q30*) What type of transport was performed by the transport company?   - - By road with a big truck (more than 6 horses)   - By road with a small truck (less than 6 horses)   - By sea   - By plane | |
| Q32. (*If it was answered "Non-commercial transportation" to Q30*) What type of vehicle did you use to transport the horse to the airport?   - - Horse trailer/float   - Goose neck   - Truck   - Two horse van - Other (please specify) ____________ | |
| Q33. (*If it was answered "Non-commercial transportation" to Q30*) Describe the traffic flow during the journey to the airport/quarantine stable   - There was no other traffic - Traffic flows were light (few stops and slow-moving traffic) - Traffic flows were moderate (some stops and slow-moving traffic) - Traffic flows were heavy (frequent stops and slow-moving traffic) | |
| Q34. Did the horse have to go to a quarantine facility?   - Yes - No | |
| Q35. (*If it was answered "Yes" to Q34*) How was the horse transported to the quarantine facility?   - - Transport company   - Non-commercial transportation | |
| Q36. (*If it was answered "Yes" to Q34*) How far is the quarantine from the airport? (In km, please) ____________ | |
| Q37. (*If it was answered "Yes" to Q34*) How long was the horse in quarantine? (In days, please) ____________ | |
| Q38. (*If it was answered "Yes" to Q34*) How many horses were in the same quarantine barn, at the same time? ____________ | |
| Q39. (*If it was answered "Yes" to Q34*) Were these horses destined for the same shipment?   - Yes - No | |
| Q40. (*If it was answered "Yes" to Q34*) Did these horses come from the same stable or origin?   - Yes - No | |
| Q41. (*If it was answered "Yes" to Q34*) During quarantine did the horse get any exercise?   - - No - Yes, walking in hand   - Yes, horse walker   - Yes, loose in a paddock   - Yes, riding under saddle - Yes, other (please specify) ____________ | |
| Q42. (*If it was answered "Yes" to Q34 and Q41*) If yes, what frequency and how long? (e.g., 1 / day for 30 minutes) ____________ | |
| Q43. Name of the Airline-Air Cargo Company the horse is traveling ____________ | |
| Q44. Arrival at the departure airport (time in hh:mm, please) ____________ | |
| Q45. Flight number ____________ | |
| Q46. Was the horse fit for transport?   - Yes - No | |
| Q47. Who did assess the fitness for transport?   - - A veterinarian   - A non-veterinarian - None | |
| **BLOCK 3 – Questions for flight groom or veterinarian at departure airport** | |
| Q48. What is your role?   - - Flight groom - Veterinarian | |
| Q49. How many years of experience do you have with handling horses? ____________ | |
| Q50. Date of departure (in dd/mm/yyyy, please) ­­­____________ | |
| Q51. External temperature at the departure airport (in degrees Celsius, please) ____________ | |
| Q52. Flight departure time (time in hh:mm, please) ____________ | |
| Q53. How many hours have the horses been in the stall before loading into the cargo? (Write a number, please) ____________ | |
| Q54. Demeanor of the horse   - - Nervous, over-responsive   - Bright, alert, responsive   - Quiet, alert, responsive   - Non-responsive - Sedated | |
| Q55. Body condition score of the horse   - - 1   - 2   - 3   - 4 - 5 | |
| Q56. Mucous membrane color of the horse   - - Pink - Pale pink - Red - Dark - Toxic line | |
| Q57. Capillary refill time of the horse (s) ____________ | |
| Q58. Heart rate of the horse (beat per minute) ____________ | |
| Q59. Respiratory rate of the horse (breath per minute) ____________ | |
| Q60. Temperature of the horse (in degrees Celsius, please) ____________ | |
| Q61. Nostril discharge   - - Unilateral   - Bilateral - None | |
| Q62. (*If it was answered "Unilateral" or “Bilateral” to Q62*) Nostril discharge type   - - Watery   - Yellowish mucus   - Greenish mucus   - Bloody mucus   - Stinky mucus | |
| Q63. Did the horse cough during the exam?   - Yes - No | |
| Q64. Were any injuries present on the horse?   - Yes - No | |
| Q65. (*If it was answered "Yes” to Q64*) What type of injury occurred?   - Shallow cut or wound - Deep cut or wound - Fracture or broken bone - Injury to hoof - Bruise/swollen part - Skin or tail rubbed raw - Other (please specify) ____________ | |
| Q66. (*If it was answered "Yes” to Q64*) What part of the horse was injured?   - - Front leg   - Back leg   - Head   - Neck   - Tail   - Chest   - Pelvis   - Abdomen   - Other (please specify) ____________ | |
| Q67. (*If it was answered "Yes” to Q64*) Reason for the injury   - - Horse Behavior (please specify) ____________   - Handling related (please specify) ____________   - Material related (please specify) ____________   - Neighbor horse behavior (please specify) ____________   - Other (please specify) ____________   - I don’t know | |
| Q68. Observe the horse for one minute and put 1 if you saw the behavior and 0 if you did not see the behavior | |
| Pawing  Tail swishing  Head tossing  Interaction with the groom  Interaction with another horse  Weaving  Vocalizing  Stamping  Rest (horse calm and relaxed)  Licking/Chewing  Sniffing  Turning the head  Eating  Other (please specify) | ○ 1 ○ 0  ○ 1 ○ 0  ○ 1 ○ 0  ○ 1 ○ 0  ○ 1 ○ 0  ○ 1 ○ 0  ○ 1 ○ 0  ○ 1 ○ 0  ○ 1 ○ 0  ○ 1 ○ 0  ○ 1 ○ 0  ○ 1 ○ 0  ○ 1 ○ 0  ○ 1 ○ 0 |
| Q69. Temperature inside the stall after loading horses (in degrees Celsius, please) ____________ | |
| Q70. Humidity inside the stall after loading horses (%) ____________ | |
| Q71. How did the horse load into the stall?   - - Easily (< 2 s)   - With a bit of hesitation (< 5 s)   - With a bit of problems (grooms had to push him)   - With lots of problems (refuse to load, slow downing the process) | |
| **BLOCK 4 – Questions for flight groom or veterinarian at the end of the journey (1 hour before landing at destination)** | |
| Q72. What is your role?   - - Flight groom   - Veterinarian | |
| Q73. Aircraft type and model number ____________ | |
| Q74. Type of flight   - - Combined flight (passenger/freight)   - Scheduled freighter flight   - Special equine charter flight (horses only) | |
| Q75. Temperature in the cargo space (in degrees Celsius, please) ____________ | |
| Q76. Humidity in the cargo space (%) ____________ | |
| Q77. Number and type of jet stall ____________ | |
| Q78. Temperature inside the stall (in degrees Celsius, please) ____________ | |
| Q79. Humidity inside the stall (%)____________ | |
| Q80. Positioning of the horse in the jet stall in the aircraft   - - Before wings   - In between wings   - After wings | |
| Q81. Estimation of total amount of cargo space used on the flight (from 0 to 100%) ____________ | |
| Q82. How many horses are traveling in this cargo? ____________ | |
| Q83. Type of air cargo stall the horse traveled   - - 1 horse   - 2 horses   - 3 horses   - Other (please specify) ____________ | |
| Q84. Type of bedding used in the jet stall   - - Straw   - Shavings   - None   - Other (please specify) ____________ | |
| Q85. How often did the horse receive water?   - - Ad libitum (always available)   - Every two hours   - Every four hours   - Not provided   - Other (please specify) ____________ | |
| Q86. Did the horse drink during the journey?   - - Yes (approximately how many liters did the horse drink during the whole flight?) ____________ - No | |
| Q87. How often did the horse receive food?   - - Ad libitum (always available)   - Every two hours   - Every four hours   - Not provided - Other (please specify) ____________ | |
| Q88. What type of food was offered?   - Grass hay - Lucerne hay - Concentrate - Other (please specify) ____________ | |
| Q89. Where was the food?   - - On the floor   - At nostril level   - At knee level - Other (please specify) ____________ | |
| Q90. Did the horse eat during the journey?   - - Yes (approximately how many kilograms did the horse eat during the whole flight?) ____________ - No | |
| Q91. Demeanor of the horse  ○ Nervous, over-responsive  ○ Bright, alert, responsive  ○ Quiet, alert, responsive  ○ Non-responsive  ○ Sedated | |
| Q92. Mucous membrane color of the horse  ○ Pink  ○ Pale pink  ○ Red  ○ Dark  ○ Toxic line | |
| Q93. Capillary refill time of the horse (s) ____________ | |
| Q94. Heart rate of the horse (beat per minute) ____________ | |
| Q95. Respiratory rate of the horse (breath per minute) ____________ | |
| Q96. Nostril discharge   - - Unilateral   - Bilateral   - None | |
| Q97. (*If it was answered "Unilateral" or “Bilateral” to Q96*) Nostril discharge type   - - Watery   - Yellowish mucus   - Greenish mucus   - Bloody mucus   - Stinky mucus | |
| Q98. Did the horse cough during the exam?   - Yes - No | |
| Q99. Were any injuries present on the horse?   - Yes - No | |
| Q100. (*If it was answered "Yes” to Q99*) What type of injury occurred?   - Shallow cut or wound - Deep cut or wound - Fracture or broken bone - Injury to hoof - Bruise/swollen part - Skin or tail rubbed raw - Other (please specify) ____________ | |
| Q101. (*If it was answered "Yes” to Q99*) What part of the horse was injured?   - - Front leg   - Back leg   - Head   - Neck   - Tail   - Chest   - Pelvis   - Abdomen   - Other (please specify) ____________ | |
| Q102. (*If it was answered "Yes” to Q99*) Reason for the injury   - - Horse Behavior (please specify) ____________   - Handling related (please specify) ____________   - Material related (please specify) ____________   - Neighbor horse behavior (please specify) ____________   - Other (please specify) ____________   - I don’t know | |
| Q103. Observe the horse for one minute and put 1 if you saw the behavior and 0 if you did not see the behavior | |
| Pawing  Tail swishing  Head tossing  Interaction with the groom  Interaction with another horse  Weaving  Vocalizing  Stamping  Rest (horse calm and relaxed)  Licking/Chewing  Sniffing  Turning the head  Eating  Other (please specify) | ○ 1 ○ 0  ○ 1 ○ 0  ○ 1 ○ 0  ○ 1 ○ 0  ○ 1 ○ 0  ○ 1 ○ 0  ○ 1 ○ 0  ○ 1 ○ 0  ○ 1 ○ 0  ○ 1 ○ 0  ○ 1 ○ 0  ○ 1 ○ 0  ○ 1 ○ 0  ○ 1 ○ 0 |
| Q104. How did the horse travel for the majority of the journey?   - - Untied   - Cross-tied   - Tied short from one side (able to move the head around and lower the head to wither height)   - Tied long from one side (able to move the head around and lower the head to knee height) - Other (please specify) ____________ | |
| Q105. Did the horse wear any shipping supplies?   - Bell boots - Rug/Cover - Travel boots - Bandages - Tail bandage - None - Other (please specify) ____________ | |
| Q106. Was the horse sedated during the journey?   - - Yes (please, indicate the type of treatment) ____________ - No | |
| Q107. Did the horse travel close to a nervous horse?   - - Yes - No | |
| Q108. Did the horse require any treatment?   - - Yes - No | |
| Q109. (*If it was answered "Yes” to Q108*) Please, describe the reason why, who did the treatment and what was administered ____________ | |
| Q110. Which one of the following scores will best describe the horse’s behavior during the journey?   - - 1 (i.e., horse pawed, scrambled, sweated, vocalized frequently, did not eat or drink)   - 2 (i.e., horse pawed, scrambled, sweated, vocalized frequently)   - 3 (i.e., the horse was agitated at the beginning of the journey but settled in after a few hours)   - 4 (i.e., the horse was agitated at the beginning of the journey but settled in soon, ate and drank regularly)   - 5 (i.e., the horse was quiet, ate and drank regularly, often rested in three legs position or slept) - Any other comments ____________ | |
| Q111. Were there any problems or delays during shipping?   - - Yes (please, explain where, how long was the delay and why it happened) ____________ - No | |
| Q112. Were there any stops during the flight?   - - Yes - No | |
| Q113. (*If it was answered "Yes” to Q112*) How many stops? ____________ | |
| Q114. (*If it was answered "Yes” to Q112*) Where were the stops? ____________ | |
| Q115. (*If it was answered "Yes” to Q112*) How long were the stops? (In hours, please) ____________ | |
| Q116. (*If it was answered "Yes” to Q112*) Were the horses unloaded from the cargo during the stops?   - - Yes - No | |
| Q117. (*If it was answered "Yes” to Q112*) Were the horses unloaded from the jet stall during the stops?   - - Yes - No | |
| Q118. (*If it was answered "Yes” to Q112*) What was the environmental temperature at the transit airport? (In degrees Celsius, please) ____________ | |
| Q119. (*If it was answered "Yes” to Q112*) What was the temperature inside the aircraft during the rest stops? (In degrees Celsius, please) ____________ | |
| **BLOCK 5 – Questions for flight/receiving groom/quarantine manager/veterinarian at arrival quarantine/destination stall** | |
| Q120. What is your role?   - - Owner   - Veterinarian - Receiving groom - Quarantine officer - Other (specify) | |
| Q121. External temperature (in degrees Celsius, please) ____________ | |
| Q122. External humidity (%) ____________ | |
| Q123. Date at arrival (in dd/mm/yyyy, please) ­­­____________ | |
| Q124. Country of arrival ____________ | |
| Q125. Time zone difference (in hours, please) ____________ | |
| Q126. Duration of journey in total (in hours, please) ____________ | |
| Q127. Were there any delays?   - - Yes (please, specify how many hours delay) ____________ - No | |
| Q128. (*If it was answered "Yes” to Q127*) What was the cause of the delay?   - - Airplane failure (please specify) ____________   - Weather (please specify) ____________ - Other (please specify) ____________ | |
| Q129. Demeanor of the horse  ○ Nervous, over-responsive  ○ Bright, alert, responsive  ○ Quiet, alert, responsive  ○ Non-responsive  ○ Sedated | |
| Q130. Body weight of the horse (in kg, please) ____________ | |
| Q131. Mucous membrane color of the horse   - - Pink - Pale pink - Red - Dark - Toxic line | |
| Q132. Heart rate of the horse (beat per minute) ____________ | |
| Q133. Respiratory rate of the horse (breath per minute) ____________ | |
| Q134. Temperature of the horse (in degrees Celsius, please) ____________ | |
| Q135. Defecation   - Hard dropping - Normal   - Not formed (cow manure consistency)   - Diarrhea | |
| Q136. Nostril discharge   - - Unilateral   - Bilateral - None | |
| Q137. (*If it was answered "Unilateral" or “Bilateral” to Q136*) Nostril discharge type   - - Watery   - Yellowish mucus   - Greenish mucus   - Bloody mucus   - Stinky mucus | |
| Q138. Lung Auscultation   - - Normal   - Abnormal - Not taken | |
| Q139. Gut sound from 0 to 3 (0 =no sound; 1 =lower motility; 2= normal motility; 3= higher motility)   - Left dorsal ____________ - Left ventral ____________ - Right dorsal ____________ - Right ventral ____________ | |
| Q140. Did the horse cough during the exam?   - Yes - No | |
| Q141. Were any injuries present on the horse?   - Yes - No | |
| Q142. (*If it was answered "Yes” to Q141*) What type of injury occurred?   - Shallow cut or wound - Deep cut or wound - Fracture or broken bone - Injury to hoof - Bruise/swollen part - Skin or tail rubbed raw - Other (please specify) ____________ | |
| Q143. (*If it was answered "Yes” to Q141*) What part of the horse was injured?   - - Front leg   - Back leg   - Head   - Neck   - Tail   - Chest   - Pelvis   - Abdomen   - Other (please specify) ____________ | |
| Q144. (*If it was answered "Yes” to Q141*) Reason for the injury   - - Horse Behavior (please specify) ____________   - Handling related (please specify) ____________   - Material related (please specify) ____________   - Neighbor horse behavior (please specify) ____________   - Other (please specify) ____________   - I don’t know | |
| Q145. Observe the horse for one minute and put 1 if you saw the behavior and 0 if you did not see the behavior | |
| Pawing  Tail swishing  Head tossing  Interaction with the groom  Interaction with another horse  Weaving  Vocalizing  Stamping  Rest (horse calm and relaxed)  Licking/Chewing  Sniffing  Turning the head  Eating  Other (please specify) | ○ 1 ○ 0  ○ 1 ○ 0  ○ 1 ○ 0  ○ 1 ○ 0  ○ 1 ○ 0  ○ 1 ○ 0  ○ 1 ○ 0  ○ 1 ○ 0  ○ 1 ○ 0  ○ 1 ○ 0  ○ 1 ○ 0  ○ 1 ○ 0  ○ 1 ○ 0  ○ 1 ○ 0 |
| Q146. Did the horse develop any diseases during the journey?   - - None   - Fever   - Diarrhea   - Colic   - Heatstroke   - Muscle problems   - Death/Euthanasia - Other ____________ | |
| **BLOCK 6 – Questions for quarantine officer/veterinarian or owner/agent one day after the journey** | |
| Q147. What is your role?   - - Owner   - Quarantine officer   - Veterinarian - Agent | |
| Q148. Demeanor of the horse   - Nervous, over-responsive - Bright, alert, responsive - Quiet, alert, responsive - Non-responsive - Sedated | |
| Q149. Body weight of the horse (in kg, please) ____________ | |
| Q150. Body condition score of the horse   - 1 - 2 - 3 - 4 - 5 | |
| Q151. Mucous membrane color of the horse   - - Pink - Pale pink - Red - Dark - Toxic line | |
| Q152. Capillary refill time of the horse (s) ____________ | |
| Q153. Heart rate of the horse (beat per minute) ____________ | |
| Q154. Respiratory rate of the horse (breath per minute) ____________ | |
| Q155. Temperature of the horse (in degrees Celsius, please) ____________ | |
| Q156. Defecation   - Hard dropping - Normal   - Not formed (cow manure consistency)   - Diarrhea | |
| Q157. Nostril discharge   - - Unilateral   - Bilateral   - None | |
| Q158. (*If it was answered "Unilateral" or “Bilateral” to Q157*) Nostril discharge type   - - Watery   - Yellowish mucus   - Greenish mucus   - Bloody mucus   - Stinky mucus | |
| Q159. Lung Auscultation   - - Normal   - Abnormal   - Not taken | |
| Q160. Gut sound from 0 to 3 (0 =no sound; 1 =lower motility; 2= normal motility; 3= higher motility)   - Left dorsal ____________ - Left ventral ____________ - Right dorsal ____________ - Right ventral ____________ | |
| Q161. Did the horse cough during the exam?   - Yes - No | |
| Q162. Were any injuries present on the horse?   - Yes - No | |
| Q163. (*If it was answered "Yes” to Q162*) What type of injury occurred?   - Shallow cut or wound - Deep cut or wound - Fracture or broken bone - Injury to hoof - Bruise/swollen part - Skin or tail rubbed raw - Other (please specify) ____________ | |
| Q164. (*If it was answered "Yes” to Q162*) What part of the horse was injured?   - - Front leg   - Back leg   - Head   - Neck   - Tail   - Chest   - Pelvis   - Abdomen   - Other (please specify) ____________ | |
| Q165. (*If it was answered "Yes” to Q162*) Reason for the injury   - - Horse Behavior (please specify) ____________   - Handling related (please specify) ____________   - Material related (please specify) ____________   - Neighbor horse behavior (please specify) ____________   - Other (please specify) ____________   - I don’t know | |
| Q166. Observe the horse for one minute and put 1 if you saw the behavior and 0 if you did not see the behavior | |
| Pawing  Tail swishing  Head tossing  Interaction with the groom  Interaction with another horse  Weaving  Vocalizing  Stamping  Rest (horse calm and relaxed)  Licking/Chewing  Sniffing  Turning the head  Eating  Other (please specify) | ○ 1 ○ 0  ○ 1 ○ 0  ○ 1 ○ 0  ○ 1 ○ 0  ○ 1 ○ 0  ○ 1 ○ 0  ○ 1 ○ 0  ○ 1 ○ 0  ○ 1 ○ 0  ○ 1 ○ 0  ○ 1 ○ 0  ○ 1 ○ 0  ○ 1 ○ 0  ○ 1 ○ 0 |
| Q167. Did the horse develop any diseases after travel?   - No - Fever - Equine transport pneumonia (i.e., shipping fever/travel sickness) - Respiratory diseases (i.e., any inflammation of the respiratory tract without fever) - Gastric ulceration - Colic - Enterocolitis - Diarrhea - Heatstroke - Muscle problems - Urinary tract problems - Laminitis - Death - Other | |
| Q168. (*If any diseases were indicated in Q167*) Please add a description of the case (e.g., type of disease, who made the diagnosis, treatment, blood and other diagnostic results, recovery) ____________ | |
| Q169. (*If it was answered "Death” to Q167*) Was a necroscopy performed?   - - No - Yes (please, describe the results of the necroscopy and the cause of death) ____________ | |
| **BLOCK 7 – Questions for quarantine officer/veterinarian or owner/agent 5 days after the journey** | |
| Q170. What is your role?   - - Owner   - Quarantine officer   - Veterinarian   - Agent | |
| Q171. Demeanor of the horse   - Nervous, over-responsive - Bright, alert, responsive - Quiet, alert, responsive - Non-responsive - Sedated | |
| Q172. Body weight of the horse (in kg, please) ____________ | |
| Q173. Body condition score of the horse   - 1 - 2 - 3 - 4 - 5 | |
| Q174. Mucous membrane color of the horse   - - Pink - Pale pink - Red - Dark - Toxic line | |
| Q175. Capillary refill time of the horse (s) ____________ | |
| Q176. Heart rate of the horse (beat per minute) ____________ | |
| Q177. Respiratory rate of the horse (breath per minute) ____________ | |
| Q178. Temperature of the horse (in degrees Celsius, please) ____________ | |
| Q179. Defecation   - Hard dropping - Normal   - Not formed (cow manure consistency)   - Diarrhea | |
| Q180. Nostril discharge   - - Unilateral   - Bilateral   - None | |
| Q181. (*If it was answered "Unilateral" or “Bilateral” to Q180*) Nostril discharge type   - - Watery   - Yellowish mucus   - Greenish mucus   - Bloody mucus   - Stinky mucus | |
| Q182. Lung Auscultation   - - Normal   - Abnormal   - Not taken | |
| Q183. Gut sound from 0 to 3 (0 =no sound; 1 =lower motility; 2= normal motility; 3= higher motility)   - Left dorsal ____________ - Left ventral ____________ - Right dorsal ____________ - Right ventral ____________ | |
| Q184. Did the horse cough during the exam?   - Yes - No | |
| Q185. Were any injuries present on the horse?   - Yes - No | |
| Q186. (*If it was answered "Yes” to Q185*) What type of injury occurred?   - Shallow cut or wound - Deep cut or wound - Fracture or broken bone - Injury to hoof - Bruise/swollen part - Skin or tail rubbed raw - Other (please specify) ____________ | |
| Q187. (*If it was answered "Yes” to Q185*) What part of the horse was injured?   - - Front leg   - Back leg   - Head   - Neck   - Tail   - Chest   - Pelvis   - Abdomen   - Other (please specify) ____________ | |
| Q188. (*If it was answered "Yes” to Q185*) Reason for the injury   - - Horse Behavior (please specify) ____________   - Handling related (please specify) ____________   - Material related (please specify) ____________   - Neighbor horse behavior (please specify) ____________   - Other (please specify) ____________   - I don’t know | |
| Q189. Observe the horse for one minute and put 1 if you saw the behavior and 0 if you did not see the behavior | |
| Pawing  Tail swishing  Head tossing  Interaction with the groom  Interaction with another horse  Weaving  Vocalizing  Stamping  Rest (horse calm and relaxed)  Licking/Chewing  Sniffing  Turning the head  Eating  Other (please specify) | ○ 1 ○ 0  ○ 1 ○ 0  ○ 1 ○ 0  ○ 1 ○ 0  ○ 1 ○ 0  ○ 1 ○ 0  ○ 1 ○ 0  ○ 1 ○ 0  ○ 1 ○ 0  ○ 1 ○ 0  ○ 1 ○ 0  ○ 1 ○ 0  ○ 1 ○ 0  ○ 1 ○ 0 |
| Q190. Did the horse develop any diseases after travel?  □ No  □ Fever  □ Equine transport pneumonia (i.e., shipping fever/travel sickness)  □ Respiratory diseases (i.e., any inflammation of the respiratory tract without fever)  □ Gastric ulceration  □ Colic  □ Enterocolitis  □ Diarrhea  □ Heatstroke  □ Muscle problems  □ Urinary tract problems  □ Laminitis  □ Death  □ Other | |
| Q191. (*If any diseases were indicated in Q190*) Please add a description of the case (e.g., type of disease, who made the diagnosis, treatment, blood and other diagnostic results, recovery) ____________ | |
| Q192. (*If it was answered "Death” to Q190*) Was a necroscopy performed?   - - No   - Yes (please, describe the results of the necroscopy and the cause of death) ____________ | |

Options for questions that had a circular bullet were a single choice, while those that had a square bullet were multiple choice.

**Table S2.** Names, definitions, data handling and categories of all the retained categorical variables.

| **Variable name** | **Definition** | **Data handling** | **Categories** |
| --- | --- | --- | --- |
| **Air journey details** | | | |
| Journey season | The season (Northern hemisphere) in which the air journey occurred. | Categorized based on the date of departure (Q50) | Winter (22 December-20 March), Spring (21 March-21 June), Summer (22 June-22 September), Autumn (23 September-21 December) |
| Flight type | Describes the type of flight, distinguishing between flights for freight, horses only or horses and passengers. | - | Combined flight (passenger/freight), Scheduled freighter flight, Special equine charter flight (horses only) |
| Country of departure | The country from which the horse started its air journey. | - | Belgium, Germany, United Kingdom, Netherlands, USA |
| Country of arrival | The destination country of the horse after air journey. | - | USA, Japan, Australia, India, South Africa, New Zealand, Qatar |
| Stops | If there was/were stop/s during the air journey. | Recategorized from the Q112 of the standard protocol. | 0 = No / 1 = Yes |
| Number of stops | If there was/were stop/s, the number of stops. | - | 0, 1, 2, 3 |
| Stop(s) unloading from cargo * | If the horse was unloaded from the cargo hold during the stop(s). | Separated for each stop from Q116 of the standard protocol question. | Yes / No |
| Stop(s) unloading from jet stall * | If the horse was unloaded from the jet stall during the stop(s). | Separated for each stop from Q117 of the standard protocol question. | Yes / No |
| Shipping delay(s) | If there was/were delay/s during the flight. | - | Yes / No |
| **Owner details** | | | |
| Relationship with the horse | If the owner was involved as a professional or amateur in the equine industry sector. | - | Professional, Amateur, Unknown, Other |
| Owner’s equestrian sector | The equestrian field in which the owner/trainer carried out horse-related activities. | - | Thoroughbred racing, Standard racing, Breeding (i.e., Thoroughbred, Warmblood, Akhal-Teke, Sport horses), Showjumping, Dressage, Eventing, Showing (i.e., Akhal-Teke), Endurance, Western sport and performance, Pony club, Recreational riding, Unknown, Other (i.e., transit stabling) |
| **Horse details** | | | |
| Horse temperament profile | Horse temperament according to positive and negative traits. | Recategorized from the temperament profile questions (Q12 of the standard protocol). If positive temperament traits prevailed, the horse was considered to have a “Good” temperament (i.e., playfulness, friendliness, understanding); conversely (i.e., nervousness/excitability, stubbornness), it was considered to have a “Bad” temperament. If temperament traits were unknown, they were categorized as “Unknown”. | Bad (i.e., nervousness/excitability, stubbornness), Good (i.e., playfulness, friendliness, understanding), Unknown |
| Horse experience in road travel | How accustomed the horse was to travel by road at the time of the air journey. | Recategorized from Q11 of the standard protocol. Horses who had no, or little travel experience were considered in the "few trips" category, while those who traveled habitually were in "Travel regularly" category. If the horse's past travel history was unknown it was included in the "Unknown" category. | Few trips (i.e., First trip, Few trips), Travel regularly (i.e., Travel regularly anxious, Travel regularly calmy), Unknown |
| Horse training to travel | The horse's training background for travel. | Recategorized from Q7 of the standard protocol. A horse trained by any method was considered "Trained", while if untrained or with an unknown history of training to travel it was considered "Untrained" or "Unknown", respectively. | Untrained (i.e., No specific transport-related training), Trained (i.e., Trained by an unknown method, Self-loading, Habituation, Positive reinforcement and negative punishment, Negative reinforcement and positive punishment), Unknown |
| Horse loading in a vehicle | Ease of loading the horse into a vehicle. | Recategorized from the Q13 of the standard protocol. If the horse showed any form of resistance to the load it was considered "Resistant," while it was "Easy" to load if it showed no signs of resistance. If this information was unknown it was considered in the "Unknown" category. | Easy, Resistant (i.e., Moderate resistant, Resistant, Highly resistant), Unknown (i.e., Never loaded, Unknown) |
| Horse previous transport-related problem behaviors (1) | If any behavioral problems related to transport were shown in the past. | - | Yes (e.g., kicking, anxiety, refusal to load), No, Unknown |
| Horse previous transport-related health problems (2) | If any health problems related to transport were manifested in the past. | - | Yes (i.e., Colic, Injury/Muscular problem), No, Unknown |
| Treatment before air journey | Treatments performed one week before the horses reached the departure quarantine | - | Yes (e.g., antibiotics, shoe removal), No, Unknown |
| Horse breed | Breed of the horse. | Recategorized form the Q21 of the standard protocol. Breeds with a frequency of less than 5% were merged into the category "Other". | Thoroughbred, Standardbred, Warmblood, Arab, Quarter Horse, Other (Irish Sport Horse, Pony, Andalusian, Akhal-Teke, Draft horse, Friesian, Spanish Purebred) |
| Horse sex | The sex of the horse. | Recategorized from the Q19 of the standard protocol. Both whole and gelded horses were included in the males. | Female (Mare, Pregnant mare), Male (Stallion, Gelding) |
| Mare in foal | Whether the female horse was pregnant. | - | Yes / No |
| Country of stable of departure | The country where the horse departure stable was located | Recategorized form the Q26 of the standard protocol. Countries with a frequency of less than 5% were merged into the category "Other". | United Kingdom, Belgium, USA, Germany, Other (Netherlands, Ireland, Canada, Czech, France, Italy, Ukraine) |
| Reason for moving the horse by air | Reason why the owner/trainer decided to transport the horse by air. | - | Racing, Competition, Sale, Breeding, Personal relocation, Other |
| **Air transport practices** | | | |
| Transport towards quarantine | How the horse reached the quarantine facility. | - | Transport company, Non-commercial transportation |
| Quarantine | If the horse stayed in the quarantine | Extrapolated from the variable “Quarantine duration (days)”. | Yes = permanence in quarantine for more than 1 day / No = permanence in quarantine for less than 1 day |
| Exercise in quarantine | If during quarantine period, the horse was exercised. | - | Yes / No |
| Type of exercise | Type of exercise the horse performed during quarantine period. | Extrapolated from Q41 of the standard protocol. | Walking in hand, Horse walker, Loose in a paddock, Riding under saddle, Other |
| Exercise frequency per day | How many times a day the horse was exercised in quarantine. | Extrapolated from Q42 of the standard protocol. | None, Once, Twice |
| Transport towards airport | How the horse reached the airport. | - | Transport company, Non-commercial transportation |
| Type of vehicle | Type of vehicle by which the horse reached the airport | - | By road with a large truck, By road with a small truck, By sea, By plane |
| Arrival at departure airport | Time of day when the horses reached the departure airport (based on the reported time). | Extrapolated from Q44 of the standard protocol. | Morning (06:00 AM-11:59 AM), Afternoon (12:00 PM-16:59 PM), Evening (17:00 PM-20:59 PM), Night (21:00 PM-5:59 AM) |
| Supplies | If the horse did or not wear supplies before the air journey. | Categorized in presence or absence of a supply based on Q105 | Yes, No |
| Horse loading in the jet stall | Describe the ease of loading the horse into a jet stall. | - | Easy, With a bit of hesitation, With a bit of problems, With lots of problems |
| Jet stall type | Based on the number of horses that can be housed in the jet stall. | - | One-horse, Two-horse, Three-horse, Other |
| Jet stall bedding | Type of bedding used in the jet stall. | Variable obtained by combining the possible answers to Q84 in the standard protocol and what was reported in "Other" option. | Straw, Shavings, None, Other (i.e., Pellets) |
| Flight departure time | Time of day when the aircraft left (based on the reported time). | Extrapolated from Q52 of the standard protocol. | Morning (06:00 AM-11:59 AM), Afternoon (12:00 PM-16:59 PM), Evening (17:00 PM-20:59 PM), Night (21:00 PM-5:59 AM) |
| Jet stall location | Where in the cargo hold the jet stall was located. | Recategorized from Q80 of the standard protocol. The jet stall located before or after the wings were considered “At extremities”. | At extremities (Before wings, After wings), In between wings |
| Percentage of cargo hold used | The percentage of cargo space occupied. | Recategorized from Q81 of the standard protocol. | ≤80, >80 |
| Watering frequency | Times the horse was watered. | Variable obtained by combining the possible answers in Q85 standard protocol and what is reported in "other". | *Ad libitum*, Every two hours, Every four hours, Not provided, Other (i.e., Every three hours) |
| Feeding frequency | Times the horse was fed. | - | *Ad libitum*, Every two hours, every four hours, Not provided, Other |
| Food type | What type of food was given to the horse. | Variable obtained by combining the possible answers in Q88 standard protocol and what is reported in "other". | Grass hay, Lucerne hay, Concentrate, Other (i.e., Haylage) |
| Food location | At what height the horse was placed food during the flight. | - | On the floor, At nostril level, At knee level, Other (i.e., At chest level) |
| Horse drinking | Whether the horse drank. | - | Yes / No |
| Horse eating | Whether the horse ate. | - | Yes / No |
| Sedation during the flight | Whether the horse received sedation during the flight. | - | Yes / No |
| Treatment during the flight | Whether the horse received treatment(s) during the flight. | - | Yes (i.e., Electrolyte vitamins paste, Oral acepromazine) / No |
| Horse tying | How the horse was tied during the flight | Recategorized from Q104 of the standard protocol. | Tied short (i.e., Cross-tied, Tied short from one side), Tied long or untied (i.e., Untied, Tied long from one side) |
| Travel near a nervous neighbor | Whether the horse traveled near a nervous neighbor during the flight. | - | Yes / No |
| **Clinical signs, behavioral parameters, and injury** | | | |
| Behavioral score one hour before landing | An overall behavioral score given to the horse one hour before landing | - | 1 = a horse that pawed, scrambled, sweated, vocalized frequently, did not eat or drink; 2 = a horse that pawed, scrambled, sweated, and vocalized frequently; 3 = a horse that was agitated at the beginning of the journey but settled in after a few hours; 4 = a horse that was agitated at the beginning of the journey but settled in soon, ate and drank regularly; 5 = a horse that was quiet, ate and drank regularly, and often rested in three legs position or slept. |
| Body condition score * | The horse's body condition score. | - | 1, 2, 3, 4, 5 |
| Nostril side with the nasal discharge * | If the horse showed lateralized nasal discharge. | - | None, Unilateral, Bilateral |
| Nasal discharge * | If the horse showed nasal discharge. | Recategorized from the variable “Nostril side with the nasal discharge”. | 0 = None / 1 = Unilateral, Bilateral |
| Nasal discharge type * | Type of nasal discharge showed by the horse. | - | Watery, Yellowish mucus, Greenish mucus, Bloody mucus, Stinky mucus |
| Increased capillary refill time (CRT > 2 s) * | If the horse showed increased (>2 s) capillary refill. | Recategorized from the variable “Capillary refill time (s)”. | 0 = ≤2 / 1 = >2 |
| Horse demeanor * | The demeanor of the horse. | - | Nervous, Bright, Quiet, Non-responsive, Sedated |
| Abnormal demeanor * | If the horse showed abnormal demeanor. | Recategorized from the variable “Horse demeanor”. | 0 = Bright, Nervous / 1 = Quiet, Non-responsive, Sedated |
| Mucous membrane color * | Color of the oral mucous membrane of the horse. | - | Pink, Pale pink, Red, Dark, Toxic line |
| Cough * | If the horse coughed during the examination. | Recategorized from the Q63, Q98, Q140, Q161, Q184 of the standard protocol. | 0 = No / 1 = Yes |
| Abnormal defecation * | Abnormal consistency of horse’s feces. | Recategorized from the variable “Defecation type”. | 0 = Normal / 1 = Diarrhea, Not formed, Hard dropping |
| Abnormal gut sounds * | Abnormal horse’s gut sounds. | The scores given to the individual quadrants in question Q139, Q160, Q183 were combined into a single score. Considering a score of 2 as normal (0) and a score other than 2 as abnormal (1), quadrants sum value greater than 0 was considered abnormal. | 0 = 2 / 1 = 1, 3 |
| Abnormal rectal temperature (RT > 38.6°C) * | If the horse developed an abnormal rectal temperature. | Recategorized from the variable “Horse rectal temperature (°C)”. | 0 = ≤38.6 °C / 1 = >38.6 °C (Shipping fever) |
| Presence of at least one abnormal clinical sign * | Occurrence of at least one abnormal clinical sign | Variable obtained leaving 1 if at least one abnormal clinical sign (nasal discharge + increased capillary refill time + cough + abnormal horse rectal temperature >38.6 °C + abnormal defecation + abnormal gut sounds) was recorded on each horse in the examined air journey phase | 0 = Yes / 1 = No |
| Total altered health | Occurrence of at least one abnormal clinical sign during all air transport phases | Variable obtained by combining data of the variable “Presence of at least one abnormal clinical sign” in the different air transport phases. | 0 = Yes / 1 = No |
| Pawing * | The horse lifted the front leg from the ground slightly, then extended it quickly in a forward direction, and moved it backward, dragging the toe against the ground in a digging motion​ (3). | - | 0 = No / 1 = Yes |
| Tail swishing * | The horse swished the tail rapidly (4). | - | 0 = No / 1 = Yes |
| Head tossing * | The horse performed a quick rotational toss of the head, similar to a head threat (5). | - | 0 = No / 1 = Yes |
| Weaving * | The horse performed a rhythmic side-to-side swaying of the head and neck or of the entire body (5). | - | 0 = No / 1 = Yes |
| Vocalizing * | The horse emitted acoustical expressions (3). | - | 0 = No / 1 = Yes |
| Stamping * | The horse sharply struck the ground with a hoof by flexing and raising and then rapidly lowering a fore or hind leg (6). | - | 0 = No / 1 = Yes |
| Licking/chewing * | The horse opened the mouth with the extension and retraction of the tongue, performed lip smacking without tongue extension or performed lateral jaw movements involving partial opening of the lips (4). | - | 0 = No / 1 = Yes |
| Turning head | The horse turned the head and neck to the right or to the left appearing to look at it flank (7). | - | 0 = No / 1 = Yes |
| Groom interaction * | The horse reached the groom with the head and interacted with him/her (sniffing, licking, rubbing, bumping) (8). | - | 0 = No / 1 = Yes |
| Horse interaction * | The horse reached the other horses with the head and interacted with them (sniffing, licking, rubbing, bumping) (4). | - | 0 = No / 1 = Yes |
| Resting * | The horse stood inactive in a relaxed posture, with the head and neck at the withers level or lower, the eyes partly or nearly closed, and the weight bearing on three legs (one hind leg slightly flexed) (6). | - | 0 = No / 1 = Yes |
| Sniffing * | The horse sniffed around (9). | - | 0 = No / 1 = Yes |
| Eating * | The horse ingested food. | - | 0 = No / 1 = Yes |
| Injury * | If the horse got injured during the air transport phases. | Recategorized from the Q64, Q99, Q141, Q162, Q185 of the standard protocol. | 0 = No / 1 = Yes |
| Injury type * | The type of injury(ies) the horse got during the air transport phases. | - | Shallow cut or wound, Deep cut or wound, Fracture or broken bone, Injury to hoof, Bruise/swollen part, Skin or tail rubbed raw, Other |
| Injured part * | The part of the horse's body that was injured during the air transport phases. | - | Front leg, Back leg, Head, Neck, Tail, Chest, Pelvis, Abdomen, Other |

* Variable collected multiple times: Horse demeanor, Abnormal demeanor, Mucous membrane color, Nostril side with the nasal discharge, Nasal discharge, Nasal discharge type, Cough, Injury, Injury type, Injured part, all the behaviors (i.e., Pawing, Tail swishing, Head tossing, Groom interaction, Horse interaction, Weaving, Vocalizing, Stamping, Resting, Licking/chewing, Sniffing, Turning head, Eating), Presence of at least one abnormal clinical sign were collected At departure, One hour before landing, On arrival, One day after arrival, Five days after arrival; Body condition score was collected At departure, One day after arrival, Five days after arrival; Increased capillary refill time was collected At departure, One hour before landing, One day after arrival, Five days after arrival; Abnormal rectal temperature was collected At departure, On arrival, One day after arrival, Five days after arrival; Abnormal defecation, Abnormal gut sounds were collected On arrival, One day after arrival, Five days after arrival; Stop(s) unloading from cargo, Stop(s) unloading from jet stall were collected for each stop performed.

**Table S3.** Names, definitions, and data handling of all the retained numerical variables.

| **Variable name** | **Explanation** | **Data handling** |
| --- | --- | --- |
| **Journey details** | | |
| N° of horses in the cargo | Total number of horses traveling in the cargo hold (other than that monitored). | - |
| N° of horses monitored | Number of horses traveling in the cargo hold that were monitored. | Extrapolated from the number of horses monitored per flight. |
| Stop duration (h) * | Duration of the stop(s). | Separated for each stop from Q115 of the standard protocol. |
| External environmental temperature during stop(s) (°C) * | The recorded environmental temperature during the stop(s). | Separated for each stop from Q118 of the standard protocol. |
| Cargo hold temperature during stop(s) (°C) * | The recorded cargo hold temperature during the stop(s). | Separated for each stop from Q119 of the standard protocol. |
| Flight duration (h) | Total flight duration calculated based on the information of the journey. | Flight duration was calculated by adding the duration of the flight (from taking off to landing, Q126) with the duration of any stops and delays. Where information was missing, it was calculated based on departure and arrival airports, average flight durations on those routes and the air journey information (stop(s) and delay(s)). |
| Flight delay duration (h) | If there was/were delay(s) in the arrival of the flight, its/their duration. | - |
| Time zone difference (h) | Hours of difference in the time between the country of arrival and the one of departure. | - |
| **Owner details** | | |
| Owner’s experience in equine industry (years) | How many years of experience had the owner/trainer in equine industry. | The following numbers are those collected in the standard protocol and how they were handled for the purpose of reporting:  “n +” = + 5; “Multiple” = 20; “Many years” = 30 |
| N° of horses in care | How many horses the owner/trainer had in his/her stud farm at the time of standard protocol completion. | The following numbers are those collected in the standard protocol and how they were handled for the purpose of reporting:  “<5” = 4; “<10” = 9; “11-30” = 20; “50-100” = 75 |
| **Horse details** | | |
| Horse age (years) | Age of the horse. | The following numbers are those collected in the standard protocol and how they were handled for the purpose of reporting:  “> 6 months” = 1 |
| **Air transport practices** | | |
| Quarantine duration (days) | The number of days the horse was in quarantine. | - |
| N° of horses housed in the same quarantine barn | Number of horses in the same barn at the same time during quarantine period. | - |
| Exercise duration (h/day) | Hours a day the horse was exercised in quarantine (including being in the paddock all day). | Extrapolated from Q42 of the standard protocol. |
| Quarantine – airport distance (km) | Distance, in kilometers, from quarantine to departure airport. | - |
| Time in the vehicle before unloading (h) | Time the horses spent in the road vehicles before being unloaded at the departure airport. | Extrapolated by subtraction between “Time in airport before departure (h)” and “Time in jet stall before landing (h)”. |
| Experience in horse handling (years) | The experience that the people in charge of handling the horse during transport (groom/vet) had in handling horses. | The following numbers are those collected in the standard protocol and how they were handled for the purpose of reporting:  “n +” = + 5 |
| Time in the jet stall before loading (h) | Time the horses spent in the jet stalls before being loaded in the aircraft. | - |
| Time in the airport before departure (h) | Time the horses spent at the airport, from their arrival until their departure by aircraft. | Extrapolated from Q44 and Q52 of the standard protocol. |
| Water intake (L/horse) | Liters of water drunk by the horse during the air journey. | If a range was reported instead of a number, the mean values was considered. If non-integer numbers (i.e., 0.5) were reported, they were rounded (i.e., 1). |
| Food consumption (kg/horse) | Kilograms of food eaten by the horse during the air journey. | If a range was reported instead of a number, the mean values was considered. If non-integer numbers (i.e., 0.5) were reported, they were rounded (i.e., 1). |
| External environmental temperature at departure airport (°C) | The external environmental temperature at the departure airport measured in Degree Celsius. | - |
| External environmental temperature at the airport of arrival (°C) | The external environmental temperature at the airport of arrival. | - |
| External environmental humidity at the airport of arrival (%) | The external environmental humidity at the airport of arrival. | - |
| Difference in temperatures on arrival and at departure (°C) | Difference between the external environmental temperature at the airport of arrival and of departure. | Calculated by subtracting temperatures on arrival and at departure. |
| Temperature in the jet stall (°C) * | The temperature inside the jet stall. | - |
| Humidity in the jet stall (%) * | The humidity inside the jet stall after horse loading. | - |
| Temperature in the cargo hold one hour before landing (°C) | The temperature inside the cargo hold before landing. | - |
| Humidity in the cargo hold one hour before landing (%) | The humidity inside the cargo hold before landing. | - |
| Difference in temperatures between jet stall and cargo hold one hour before landing (°C) | Difference between the temperature inside the jet stall and in the cargo hold one hour before landing. | Calculated by subtracting temperatures inside the jet stall and in the cargo one hour before landing. |
| Difference in humidity between jet stall and cargo hold one hour before landing (°C) | Difference between the humidity inside the jet stall and in the cargo hold one hour before landing. | Calculated by subtracting humidities inside the jet stall and in the cargo one hour before landing. |
| Difference in temperatures in jet stall one hour before landing and at taking off (°C) | Difference between the temperatures inside the jet stall one hour before landing and at taking off. | Calculated by subtracting temperatures inside the jet stall one hour before landing and at taking off. |
| Difference in humidity in jet stall one hour before landing and at taking off (°C) | Difference between the humidity inside the jet stall one hour before landing and at taking off. | Calculated by subtracting humidity inside the jet stall one hour before landing and at taking off. |
| **Physiological parameters** | | |
| Heart rate (bpm) * | Heart rate of the horse. | “Normal” = 35 bpm |
| Respiratory rate (bpm) * | Respiratory rate of the horse. | “Normal” = 18 bpm |
| Horse rectal temperature (°C) * | Rectal temperature of the horse. | “Normal” = 37.5 °C |

* Variable collected multiple times: Heart rate (bpm) and Respiratory rate (bpm) were collected At departure, One hour before landing, On arrival, One day after arrival. Five days after arrival; Horse rectal temperature (°C) was collected At departure, On arrival, One day after arrival, Five days after arrival; Temperature in the jet stall (°C) and Humidity in the jet stall (%) were collected At departure and One hour before landing; Stop duration (h), External environmental temperature during stop(s) (°C), Cargo hold temperature during stop(s) (°C) were collected for each stop performed.

**Table S4.** Descriptive statistics of the clinical signs: nostril side with the nasal discharge, nasal discharge type, abnormal defecation, mucous membrane color, and horse demeanor. Data obtained from 118 horses undergoing air transport.

| **Variable** | **Journey phase** | **Category** | **Number** | **Percentage** |
| --- | --- | --- | --- | --- |
| Nostril side with the nasal discharge | At departure (n = 12) | Unilateral | 3 | 25% |
|  |  | Bilateral | 9 | 75% |
|  | One hour before landing (n = 29) | Unilateral | 4 | 14% |
|  |  | Bilateral | 25 | 86% |
|  | On arrival (n = 13) | Unilateral | 2 | 15% |
|  |  | Bilateral | 11 | 85% |
|  | One day after arrival | na | na | na |
|  | Five days after arrival | na | na | na |
| Nasal discharge type | At departure (n = 12) | Watery | 10 | 83% |
|  |  | Serous | 2 | 17% |
|  | One hour before landing (n=29) | Watery | 27 | 93% |
|  |  | Serous | 1 | 4% |
|  |  | Yellowish mucus | 1 | 4% |
|  | On arrival (n = 13) ^a^ | - | - | - |
|  | One day after arrival | na | na | na |
|  | Five days after arrival | na | na | na |
| Mucous membrane color | At departure (n = 91) ^a^ | Pale pink | 15 | 16% |
|  |  | Pink | 76 | 84% |
|  | One hour before landing (n = 99) ^a^ | Pale pink | 15 | 15% |
|  |  | Pink | 82 | 83% |
|  |  | Red | 2 | 2% |
|  | On arrival (n = 68) ^a^ | Pale pink | 12 | 18% |
|  |  | Pink | 56 | 82% |
|  | One day after arrival (n = 75) ^a^ | Pale pink | 2 | 3% |
|  |  | Pink | 73 | 97% |
|  | Five days after arrival (n = 70) ^a^ | Pink | 70 | 100% |
| Horse demeanor | At departure (n = 109) ^a^ | Nervous | 7 | 6% |
|  |  | Bright | 62 | 57% |
|  |  | Quiet | 38 | 35% |
|  |  | Non-responsive | 1 | 1% |
|  |  | Sedated | 1 | 1% |
|  | One hour before landing (n = 102) ^a^ | Nervous | 2 | 2% |
|  |  | Bright | 34 | 33% |
|  |  | Quiet | 65 | 64% |
|  |  | Non-responsive | 1 | 1% |
|  | On arrival (n = 68) ^a^ | Nervous | 7 | 10% |
|  |  | Bright | 43 | 63% |
|  |  | Quiet | 17 | 25% |
|  |  | Non-responsive | 1 | 1% |
|  | One day after arrival (n = 75) ^a^ | Nervous | 3 | 4% |
|  |  | Bright | 35 | 47% |
|  |  | Quiet | 37 | 49% |
|  | Five days after arrival (n=70) ^a^ | Nervous | 3 | 4% |
|  |  | Bright | 38 | 54% |
|  |  | Quiet | 29 | 41% |

Total percentages: It is important to note that total percentages may not always sum to 100% for every characteristic due to rounding.

nr: Not recorded in the standard protocol.

na: Not applicable (i.e., no altered clinical parameter).

^a^ Missing data: Data were missing for some variables, and the denominator for percentage calculations is based on the number of horses for which data was available. The total number of horses with available data is indicated next to each variable descriptor.

Data were missing for Horse demeanor (missing = 9 horses, 8%) and Mucous membrane color at departure (missing = 27 horses, 23%); Horse demeanor (missing = 16 horses, 14%) and Mucous membrane color one hour before landing (missing = 19 horses, 16%); Nasal discharge type (missing = 13 horses, 100%), Horse demeanor (missing = 50 horses, 42%) and Mucous membrane color on arrival (missing = 50, 42%); Horse demeanor (missing = 43 horses, 36%) and Mucous membrane color one day after arrival (missing = 43 horses, 36%); Horse demeanor (missing = 70 horses, 41%) and Mucous membrane color five days after arrival (missing = 48 horses, 41%).

**Table S5.** Descriptive statistics of heart rate (beats per minute), respiratory rate (breaths per minute), and horse rectal temperature (degrees Celsius) and their association with the air transport phases. Data obtained from 118 horses undergoing air transport.

| **Variable** | **Journey phase** | **Median** | **Min** | **IQR** | **Max** | **Missing** | **Friedman Rank Sum Test P value** |
| --- | --- | --- | --- | --- | --- | --- | --- |
| Heart rate (bpm) | At departure | 40 | 26 | 36-48 | 90 | 31% | **0.007** |
|  | One hour before landing | 38 | 24 | 36-40 | 60 | 32% |  |
|  | On arrival | 40 | 32 | 36-44 | 80 | 54% |  |
|  | One day after arrival | 36 | 32 | 36-40 | 52 | 60% |  |
|  | Five days after arrival | 36 | 32 | 32-36 | 44 | 63% |  |
| Respiratory rate (bpm) | At departure | 14 | 8 | 12-24 | 72 | 28% | **0.013** |
|  | One hour before landing | 14 | 10 | 12-21 | 64 | 28% |  |
|  | On arrival | 20 | 12 | 18-26 | 56 | 58% |  |
|  | One day after arrival | 16 | 8 | 14-20 | 26 | 59% |  |
|  | Five days after arrival | 16 | 8 | 14-20 | 24 | 63% |  |
| Horse rectal temperature  (°C) | At departure | 37.6 | 36.4 | 37.4-37.8 | 38.1 | 41% | na |
|  | One hour before landing | nr | nr | nr | nr | nr |  |
|  | On arrival | 37.8 | 36.9 | 37.7-38.2 | 40.7 | 43% |  |
|  | One day after arrival | 37.8 | 37.0 | 37.7-38.1 | 39.0 | 36% |  |
|  | Five days after arrival | 37.8 | 37.0 | 37.7-38.0 | 38.7 | 41% |  |

Total percentages: It is important to note that total percentages may not always sum to 100% for every characteristic due to rounding.

Friedman Rank Sum Test Significant P values (i.e., P value ≤ 0.05) are shown in bold.

nr: Not recorded in the standard protocol.

na: Not applicable due to the absence of the check during one phase

**Table S6.** List of Wald test P values obtained from the univariable logistic regression model for the outcomes of nasal discharge recorded one hour before landing.

| **Independent variable** | **Wald test P value** |
| --- | --- |
| **Air journey details** | |
| Journey season | **0.021** |
| Stops | 0.431 |
| N° of horses in the cargo | **0.008** |
| Flight duration (h) | 0.938 |
| **Horse details** | |
| Horse temperament profile | **<0.001** |
| Horse experience in road travel | 0.459 |
| Horse training to travel | 0.772 |
| Horse loading in a vehicle | 0.689 |
| Horse previous transport-related problem behaviors | 0.936 |
| Treatment before air journey | 0.336 |
| **Air transport practices** | |
| Jet stall type | 0.679 |
| Jet stall bedding | 0.448 |
| Jet stall location | **0.099** |
| Percentage of cargo hold used | 0.544 |
| Food type | **0.032** |
| Quarantine | 0.514 |
| External environmental temperature at departure airport (°C) | **0.002** |

P values < 0.100 are shown in bold as these independent variables were included in the multivariable logistic regression model.

**Table S7.** List of Wald test P values obtained from the univariable logistic regression model for the outcomes of increased capillary refill time recorded one hour before landing.

| **Independent variable** | **Wald test P value** |
| --- | --- |
| **Air journey details** | |
| Stops | **<0.001** |
| N° of horses in the cargo | **0.013** |
| Flight duration (h) | **<0.001** |
| **Horse details** | |
| Horse experience in road travel | **0.083** |
| **Air transport practices** | |
| Jet stall type | 0.122 |
| Jet stall bedding | 0.339 |
| Jet stall location | 0.532 |
| Percentage of cargo hold used | 0.487 |
| Food type | 0.122 |
| Quarantine | **<0.001** |
| Horse tying | **<0.001** |
| External environmental temperature at departure airport (°C) | 0.744 |

P values < 0.100 are shown in bold as these independent variables were included in the multivariable logistic regression model.

**Table S8.** List of the independent variables related to journey and horse details and air transport practices tested for univariable regression analyses.

| **Independent variable** |
| --- |
| **Air journey details** |
| Journey season |
| Country of departure |
| Stops |
| N° of horses in the cargo |
| Flight duration (h) |
| **Horse details** |
| Horse temperament profile |
| Horse experience in road travel |
| Horse training to travel |
| Horse loading in a vehicle |
| Horse previous transport-related problems behavior |
| Treatment before air journey |
| Horse breed |
| Horse sex |
| Country of stable of departure |
| Reason for moving the horse by air |
| Horse age (years) |
| **Air transport practices** |
| Quarantine |
| Arrival at departure airport |
| Jet stall type |
| Jet stall bedding |
| Flight departure time |
| Jet stall location |
| Percentage of cargo hold used |
| Watering frequency |
| Food type |
| Horse tying |
| Experience in horse handling (years) |
| External environmental temperature at departure airport (°C) |
| Temperature in the cargo hold one hour before landing (°C) |

**References**

1. Padalino B, Henshall C, Raidal SL, Knight P, Celi P, Jeffcott L, et al. Investigations into equine transport-related problem behaviors: survey results. J Equine Vet Sci. 2017;48:166-73. e2.
2. Benedetti B, Felici M, Thiébaud G, Freccero F, Padalino B. Survey of horse transportation in Switzerland: practices and issues. Schweiz Arch Tierheilkd. 2023;165(9):573-84.
3. McDonnell S, Haviland J. Agonistic ethogram of the equid bachelor band. Appl Anim Behav Sci. 1995;43(3):147-88.
4. Riva MG, Sobrero L, Menchetti L, Minero M, Padalino B, Dalla Costa E. Unhandled horses classified with broken/unbroken test (BUT) exhibit longer avoidance, flight reactions, and displacement behaviors when approached by humans. Front Vet Sci. 2022;9:1022255.
5. Torcivia C, McDonnell S. Equine discomfort ethogram. Animals. 2021;11(2):580.
6. McDonnell SM. The equid ethogram: a practical field guide to horse behavior: Eclipse Press; 2003.
7. Padalino B, Raidal SL, Knight P, Celi P, Jeffcott L, Muscatello G. Behaviour during transportation predicts stress response and lower airway contamination in horses. PLoS One. 2018;13(3):e0194272.
8. Padalino B, Siniscalchi M, Lusito R, Quaranta A, editors. Effect of T-Touch on behavior of Saddle Horses. Proceedings of 9th International Equitation Science Conference; 2013.
9. Padalino B, Raidal SL. Effects of transport conditions on behavioural and physiological responses of horses. Animals. 2020;10(1):160.
